# The Structure of Zinc Chelidonate in the Crystalline Phase, Aqueous Solution and Assessment of the Interaction with Serum Albumin

**DOI:** 10.3390/molecules31091378

**Published:** 2026-04-22

**Authors:** Stanislav Kozin, Victor Dotsenko, Nicolay Aksenov, Alexandr Bespalov, Alexandr Kravtsov, Oksana Lyasota, Anna Dorohova, Viacheslav Kindop, Sergei Bobrovnik, Arkady Moiseev, Lev Ivashchenko, Evgeny Gerasimenko, Tran Quang Huy, Stepan Dzhimak

**Affiliations:** 1 Physics and Technology Faculty, Kuban State University, 350040 Krasnodar, Russiatamgdenet711@mail.ru (S.B.); 2 Laboratory of Problems of Stable Isotope Spreading in Living Systems, Federal Research Center the Southern Scientific Center of the Russian Academy of Sciences, 344006 Rostov-on-Don, Russia; 3 Faculty of Chemistry and High Technologies, Kuban State University, 350040 Krasnodar, Russia; victor_dotsenko_@mail.ru (V.D.); slavakindop@mail.ru (V.K.);; 4 Faculty of Chemistry, North Caucasus Federal University, 355017 Stavropol, Russia; 5 Scientific Department, Kuban State Agrarian University, 350040 Krasnodar, Russia; 6 Department of Technology of Fats, Cosmetics, Commodity Science, Processes and Devices, Kuban State Technological University, 350072 Krasnodar, Russia; 7 Phenikaa University Nano Institute, Phenikaa School of Engineering, Phenikaa University, Hanoi 12116, Vietnam

**Keywords:** zinc, zinc complexes, chelidonic acid, 4-oxo-4H-pyrans, intrinsic protein fluorescence, bovine serum albumin

## Abstract

A zinc complex of chelidonic acid (4-oxo-4H-pyran-2,6-dicarboxylic acid) was obtained by reaction with zinc oxide under isothermal conditions. Its composition was confirmed by elemental and thermogravimetric analyses, and its molecular structure was characterized using NMR and IR spectroscopy. Single-crystal X-ray diffraction revealed that the complex crystallizes as a one-dimensional coordination polymer, [ZnChel(H_2_O)_4_]_n_, in the triclinic space group P-1, featuring a distorted octahedral Zn(II) center coordinated by two chelidonate ligands and four water molecules. This six-coordinate arrangement contrasts with previously described tetra-coordinated Zn–chelidonate complexes. Quantum-chemical calculations and molecular dynamics simulations indicated that, in aqueous solution, Zn(II) preferentially forms a monodentate ZnChel(H_2_O)_5_ species, consistent with the solid-state coordination environment. The interaction of the complex with bovine serum albumin (BSA) was examined by fluorescence, UV–Vis absorption, and circular dichroism spectroscopy, revealing a mixed static–dynamic quenching mechanism, moderate binding affinity, and hydrogen-bonding/van der Waals contributions accompanied by alterations in BSA secondary structure. These results expand the structural chemistry of chelidonic acid and provide biophysical insight into the protein-binding behavior of zinc chelidonate, supporting its potential relevance as a zinc-based bioactive compound.

## 1. Introduction

Zinc is an essential micronutrient that is present in the human body predominantly in protein-bound form. Approximately 10% of human proteins are capable of binding Zn^2+^, indicating its involvement in diverse biochemical pathways [1]. Zinc functions as a cofactor in more than 300 enzymes, including alkaline phosphatase, sphingomyelinase, carbonic anhydrase, matrix metalloproteinases, and alcohol dehydrogenase, where it contributes to stabilization of the catalytic center and participates directly in enzymatic reactions [2]. Zinc ions participate in the formation of zinc finger domains, which stabilize protein structure and regulate transcriptional activity. These include proteins of the IKAROS family, which play key roles in lymphocyte development and immune signaling [3]. Maintenance of zinc homeostasis is therefore essential for normal immune function, including hematopoiesis, cell maturation and differentiation, and cytokine production [4]. Although zinc is redox-inert, it contributes indirectly to antioxidant defense [5]. Zinc is a structural component of superoxide dismutase and stabilizes cellular membranes and protein sulfhydryl groups, thereby reducing oxidative injury [6]. In addition, Zn^2+^ induces the expression of metallothioneins, natural chelators that bind and sequester toxic metal ions [7]. Through these mechanisms, zinc modulates oxidative stress and inflammatory responses.

Zinc is a key regulator of endocrine function, and its deficiency is associated with an increased risk of metabolic disorders [8]. In pancreatic β-cells, Zn^2+^ is required for insulin synthesis, storage, and secretion [9]. Insufficient zinc intake is also linked to impaired sex hormone production, defective spermatogenesis, and male infertility [10].

In the central nervous system, zinc accumulates in synaptic vesicles, particularly in the hippocampus where it modulates NMDA receptor activity and thereby influences learning and memory [11].

Recent studies further highlight the role of Zn^2+^ in cardiovascular regulation, acting through receptors such as GPR39 and RyR2, as well as TRPC6 channels, to modulate endothelial proliferation, angiogenesis, vasodilation, and myocardial contractility [12,13]. Zinc additionally participates in all phases of wound and burn healing, while zinc deficiency contributes to delayed tissue repair and dermatological complications [14].

Zinc deficiency remains prevalent worldwide, including in economically developed countries, largely due to inadequate dietary intake and limited bioavailability. Dietary sources alone are often insufficient, as zinc absorption is reduced by components such as phytates and proteins [15]. Consequently, zinc supplementation is widely used to prevent and treat hypozincosis. However, conventional zinc preparations present important limitations. Inorganic salts such as zinc oxide and zinc sulfate display relatively poor bioavailability and gastrointestinal adverse effects, whereas organic salts, including picolinate, citrate, and gluconate may disturb the homeostasis of other essential metal ions and lack well-defined therapeutic advantages beyond supplementation. With increasing focus on targeted prevention and treatment of endocrine, neurological, cardiovascular, and other disorders associated with zinc imbalance, there is a growing need for safe and rational zinc delivery systems. In this context, chelidonic acid is proposed as a promising ligand for zinc coordination, offering the potential to enhance bioavailability while conferring additional biological activity.

Chelidonic acid (4-oxo-4H-pyran-2,6-dicarboxylic acid; H_2_Chel) is a low-molecular-weight bioactive compound belonging to the γ-pyran acid series and is a natural constituent of the medicinal plant *Chelidonium majus* L. H_2_Chel has demonstrated anti-inflammatory and antihistamine activity, including inhibition of mast-cell degranulation, a reduction in circulating eosinophils, and suppression of serum IgE levels [16,17]. In experimental models of ulcerative colitis and allergic rhinitis, it attenuates the production of pro-inflammatory mediators [18,19]. Complexation of H_2_Chel with calcium has been shown to enhance extracellular matrix mineralization in vitro and promote ectopic bone formation in situ [20]. Additional studies suggest that H_2_Chel exerts anxiolytic and anti-inflammatory effects in the central nervous system and may stimulate expression of the neurotrophic factor BDNF [21]. Upregulation of the antioxidant-responsive transcription factor Nrf2 and scavenging of peroxide radicals have also been reported, together with inhibition of the Fenton reaction [22,23,24,25]. Related pyran-derivative acids such as comenic, kojic, and meconic acids likewise exhibit diverse biological activities, further supporting the pharmacological relevance of this structural class [26,27,28,29].

The crystal structures of several coordination compounds of chelidonic acid with metal cations, including Mn^2+^, Ca^2+^, Be^2+^, Cu^2+^, and Cd^2+^, have been reported [30]. In the beryllium complex [Be(H_2_O)_4_]^2+^(C_7_H_2_O_6_)_2_, the cation is coordinated through hydrogen-bond networks involving water molecules, giving rise to a three-dimensional framework. Each of the ions in the crystal (N_2_H_6_)^2+^[Ca(C_7_H_2_O_6_)]_2_(H_2_O)_2_]_2_, lies on the crystallographic axis of the second order in the space group P2/c; anions form sheets with hydrogen bonds, which the cations link into a three-dimensional framework. In the calcium salt (N_2_H_6_)_2_[Ca(C_7_H_2_O_6_)_2_(H_2_O)_2_], which crystallizes in space group P2/c, the chelidonate anions form hydrogen-bonded layers that are further linked into a three-dimensional architecture by the cations. The manganese tetrahydrate [Mn_2_(C_7_H_2_O_6_)_2_(H_2_O)_8_]·4H_2_O consists of centrosymmetric Mn_2_(C_7_H_2_O_6_)_2_ units connected through an extensive hydrogen-bonding network. The copper complex [Cu(C_7_H_2_O_6_)(H_2_O)_4_] contains two crystallographically independent Cu^2+^ ions located on inversion centers in space group P-1, forming a one-dimensional coordination polymer further assembled into a three-dimensional framework via hydrogen bonding. In contrast, the magnesium chelidonate complex features a Mg^2+^ ion hexacoordinated exclusively by water molecules, with chelidonate ligands participating in extended hydrogen-bonding interactions [31].

Several zinc–chelidonate coordination compounds have previously been reported [32,33]. In one case, the crystal structure revealed incorporation of DMSO molecules within the lattice, while in another, hydrothermal synthesis yielded a complex in which Zn(II) is coordinated by two chelidonate ligands and two water molecules, giving a tetra-coordinated environment.

Despite the biological relevance of chelidonic acid and the increasing number of metal–chelidonate structures, zinc chelidonate has been structurally characterized only in tetra-coordinated forms, and its behavior in aqueous solution and interaction with transport proteins such as serum albumin remain unexplored. Therefore, we synthesized and structurally characterized a new octa-coordinated Zn–chelidonate coordination polymer, [ZnChel(H_2_O)_4_]_n_; investigated its speciation in aqueous solution by quantum-chemical methods; and evaluated its binding to bovine serum albumin using multi-spectroscopic techniques.

## 2. Results and Discussion

### 2.1. Chemical Section

#### 2.1.1. Synthesis Results

The zinc chelidonate complex [Zn(Chel)(H_2_O)_4_]_n_ was synthesized via a straightforward ion-exchange reaction between chelidonic acid and zinc oxide in an aqueous medium at 60 ± 2 °C. Chelidonic acid (0.45 g, 2.4 mmol) was dissolved in 15 mL of water, and zinc oxide (0.26 g, 1.2 mmol) was added portionwise with stirring. The reaction mixture turned yellow (pH 5.5–6.0), indicating complex formation. Upon evaporation to approximately 75% of the original volume, crystallization occurred. The pale crystals were isolated by filtration, washed with cold water, and recrystallized from bidistilled water to afford the product in 80% yield.

#### 2.1.2. NMR Spectroscopy

Figure S1 shows the ^1^H NMR spectrum of chelidonic acid recorded in D_2_O. A single resonance is observed at δ 7.11 ppm, corresponding to the two equivalent aromatic protons H-3 and H-5. Signals from the carboxylic acid protons are absent due to rapid H/D exchange in D_2_O.

Notably, the proton resonance at δ 7.11 ppm exhibits ^13^C satellite splitting, appearing as a doublet of doublets (AMX-type pattern; ^1^J_1_H–^13^C = 172.2 Hz, ^3^JH–H = 2.20 Hz). This splitting arises from the minor isotopomer containing one ^13^C nucleus at either C-3 or C-5 (Figure S2). Figure S3 present the ^13^C NMR spectra of chelidonic acid recorded in D_2_O.

In the ^13^C NMR spectrum, the resonance corresponding to C-3/C-5 is observed at δ 117.4 ppm. The assignments of the remaining carbon signals are summarized in Figure S4 and show good agreement with previously reported data [31]. Figure S5 presents the ^1^H NMR spectrum of [Zn(Chel)(H_2_O)_4_]_n_. Comparison with the corresponding spectrum of chelidonic acid (Figure S1) shows a slight upfield shift in the aromatic proton resonance, from δ 7.11 ppm to δ 7.01 ppm.

As in the free acid, the proton signal exhibits ^13^C satellite splitting in the form of a doublet of doublets (AMX system; ^1^J_1_H–^13^C = 171.9 Hz, ^3^JH–H = 2.34 Hz), which arises from the minor isotopomer containing a single ^13^C nucleus at either C-3 or C-5 (Figure S6). Overall, the ^1^H NMR spectral features of [Zn(Chel)(H_2_O)_4_]_n_ closely resemble those of chelidonic acid.

The ^13^C NMR spectrum of [Zn(Chel)(H_2_O)_4_]_n_ is shown in Figure S7. Comparison with the spectrum of chelidonic acid reveals a downfield shift in carboxyl carbon resonances from δ 163.9 to 165.2 ppm (Δδ = 1.3 ppm). This shift is consistent with deprotonation of both carboxyl groups and their participation in coordination to Zn^2+^ or coordination-assisted interactions with bound water molecules.

These observations are consistent with literature reports describing downfield displacement of carboxylate carbon resonances in ^13^C NMR spectra relative to those of protonated carboxyl groups [34]. In zinc chelidonate [Zn(Chel)(H_2_O)_4_]_n_, the pyran ring carbons C-3 and C-5 exhibit an upfield shift of Δδ = 1.1 ppm (from 117.4 to 116.3 ppm), whereas the C-2 and C-6 carbons shift downfield by Δδ = 1.9 ppm (from 157.7 to 159.6 ppm). The carbonyl carbon C-4 experiences only a minor downfield shift of Δδ = 0.7 ppm (from 184.4 to 185.1 ppm). The chemical shift changes at C-3/C-5 and C=O suggest modest alterations in the electronic environment of the chelidonate ion and support the absence of direct Zn^2+^ coordination to the ring carbonyl oxygen atoms.

The ^13^C NMR spectrum of zinc chelidonate shows a downfield shift in the carboxylate carbon signal from δ 163.9 ppm (free acid) to δ 165.2 ppm (Δδ = 1.3 ppm), which is characteristic of carboxylate anion formation following deprotonation [34]. Although rapid H/D exchange in D_2_O precludes direct observation of the carboxylic OH protons, and NMR alone cannot unambiguously prove complete deprotonation, this shift provides consistent supporting evidence. The conclusion of double deprotonation is strongly supported by charge balance (Zn^2+^ requires two Chel^2−^ ligands), X-ray structural data, and IR spectroscopic results (see below).

#### 2.1.3. IR Spectroscopy

The IR spectrum of the zinc complex lacks the characteristic C=O stretching band of protonated carboxylic groups at 1675 cm^−1^, which is clearly present in the spectrum of free chelidonic acid (Figure S8). This disappearance, along with the appearance of strong asymmetric (1582 cm^−1^) and symmetric (1355 cm^−1^) carboxylate stretching bands, indicates deprotonation of both –COOH groups. Although the broad OH stretching region (centered at 3103 cm^−1^) is dominated by coordinated water molecules and does not allow direct assessment of residual acidic OH groups, the spectroscopic pattern, combined with the required charge balance, the observed ^13^C NMR carboxylate shift, and the X-ray confirmation of carboxylate coordination, unequivocally supports the presence of dianionic chelidonate ligands in the complex [20,32,35]. The coordination mode of the carboxylate group was evaluated using the frequency separation Δν = νas(COO^−^) − νs(COO^−^) [36].

For zinc chelidonate, Δν = 227 cm^−1^, a value more consistent with monodentate or bridging coordination rather than symmetric chelating, as supported by literature [37]. In addition, the presence of a broad absorption band centered at 3103 cm^−1^ indicates coordinated or lattice water molecules in the complex.

#### 2.1.4. Thermal Analysis

Analysis of the thermal behavior and composition of the [Zn(Chel)(H_2_O)_4_]_n_ hydrate (Figure S9) revealed that the thermolysis of these compounds occurs in several stages. The first weak endothermic effect at 226.2 °C with a mass loss of 10.33% is the mechanism of the removal of one coordinated water molecule from the zinc sphere. The position of the removed water molecules is likely occupied by the carboxylate groups of the ligand. A subsequent increase in temperature is accompanied by two exo-effects (92.1 μW s/mg at 263.6 and 313.7 °C) with a mass loss of 5.89 and 10.76%, respectively, which are apparently caused by further dehydration of the sample and reflect the sequential elimination of one more and then two remaining water molecules from the zinc coordination sphere. A relatively weak exo-effect (21.2 μW s/mg at 313.7 °C) is apparently caused by the incipient decarboxylation of the ligand molecules. A strong exo-effect at 410.2 °C, accompanied by a mass loss of 37.36%, is caused by thermal destruction and subsequent burnout of the ligand molecules. The end product of thermolysis is zinc oxide. According to the results of thermal analysis, the composition of zinc chelidonate coincides with the results of X-ray diffraction and elemental analysis and has the form [ZnChel(H_2_O)_4_] (Table S1).

#### 2.1.5. Elemental Analysis Data

Elemental composition data obtained using three different methods are in good agreement with X-ray diffraction data (Table 1).

#### 2.1.6. Description of X-Ray Diffraction Patterns of Zinc Chelidonate [Zn(Chel)(H_2_O)_4_]_n_ Single Crystals

**Figure 1:**
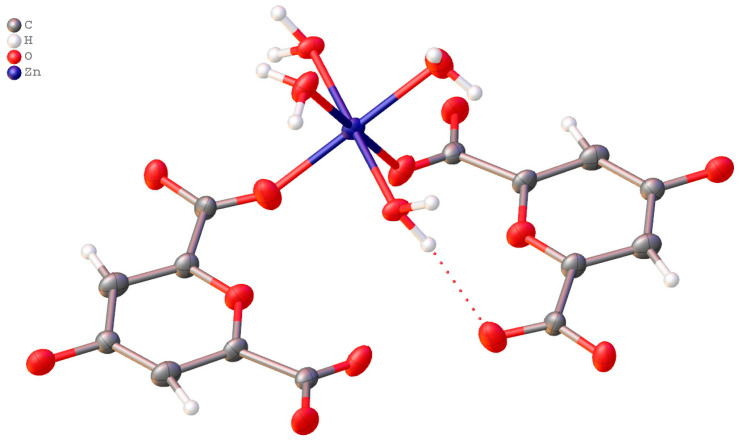
The independent region in the zinc chelidonate structure. Thermal ellipsoids are shown at 50% probability. Hydrogen bonds are depicted as dashed lines.

**Figure 2:**
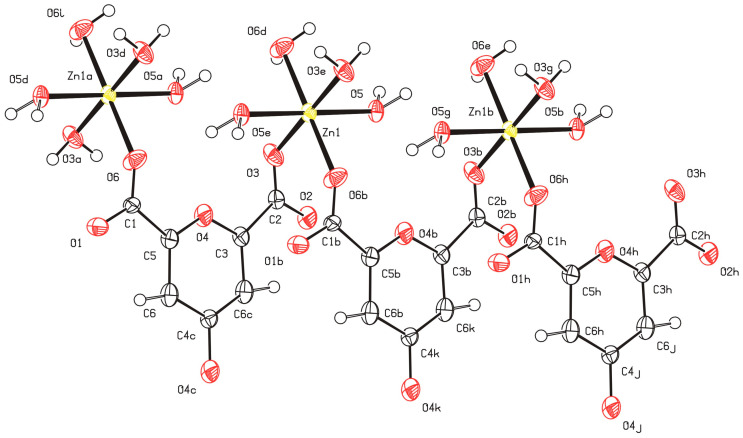
Fragment of the single-crystal structure of zinc chelidonate. Thermal ellipsoids are shown at a 50% probability.

The crystal structure of ChelZn is a one-dimensional chain structure constructed by hybridization of Chel^2−^ chelidonic acid residues with inorganic Zn(H_2_O)_4_^2+^ fragments (Figure 1 and Figure 2).

The structure is a non-merohedral twin defined by the transformation matrix (−1 0 0, 0 −1 0, 0.325 0.340 1) and was refined using the HKLF 5 format. The refinement is further complicated by the chelidonic acid fragment residing on a crystallographic inversion center. Consequently, the O4 atom statistically represents both the pyran oxygen and the carbonyl oxygen. This symmetry-imposed disorder is reflected in the partial occupancies (0.5) of atoms C1, C2, C3, C5, and O1, O2, accounting for the two superimposed molecular orientations within the lattice. In this model, the O3 and O6 positions represent either coordinated water molecules or components of the carboxylic acid fragment, depending on the local orientation of the disordered ligand. While all other atoms were reliably localized, the H6 hydrogen atom could not be precisely positioned. Its electron density is diffuse due to the superposition of orientations, and standard geometric restraints (AFIX) are inapplicable in this specific site-symmetry case. The superposition of the two chelidonic acid orientations is illustrated below.

**Figure 3:**
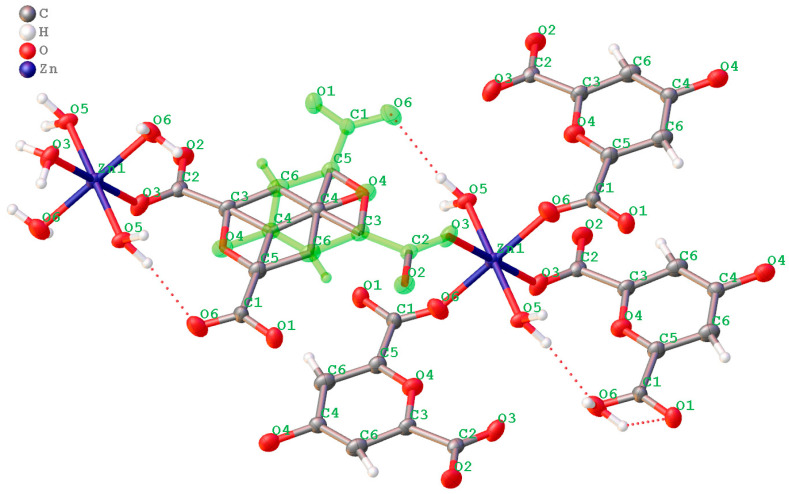
Disorder of the complex around the inversion center located between C4-C4.

The structure contains a non-merohedral twin with the transformation matrix −1 0 0/0 −1 0/0.325 0.340 1, which was treated using an HKLF 5 refinement strategy. The structure is also complicated by the presence of an inversion center within the chelidonic acid fragment. Consequently, the O4 atom can act in different parts of the crystal either as a pyran oxygen or as a carbonyl oxygen. The influence of the inversion center is reflected in the occupancies of the model, resulting in a disordered structure when represented. This disorder is not entirely standard, showing several possible arrangements of the atoms in the crystal, but rather a way of describing the structure in the presence of an element of symmetry within the molecule. Thus, carbon atoms C1, C2, C3, and C5, as well as oxygen atoms O1 and O2, have occupancies of 0.5 due to the presence or absence of carboxyl groups arising from the statistical distribution of molecular orientations in the crystal lattice. Oxygen atoms O3 and O6 in this case correspond to water molecules and are not part of the carboxylic acid fragment. Nevertheless, the positions of all non-hydrogen atoms were determined reliably, except for hydrogen H6, which should have a different spatial position depending on the opposite orientation of the ligand. However, the influence of the inversion center in the model and the weak residual electron density do not allow accurate determination of its position, and standard AFIX instructions are not designed to model such cases. A representation of the superposition of two chelidonic acid fragments is shown below (Figure 3).

The parameters of the photoelectron capture and the crystal system are presented in Tables S2–S5. The coordination geometry of the Zn ion is a slightly distorted octahedron with four oxygen atoms from two water molecules and two oxygen atoms from the Chel^2−^ carboxylate ions. The bond angles around the Zn(II) ion are in the range of 86.16–93.84°, and the Zn–O bond lengths vary from 2.064 to 2.164 Å. Thus, the coordination number of zinc is 6. There are no free water molecules in the crystal lattice, but there are four bound water molecules within the first coordination sphere of the zinc cation. Bound water molecules are equally involved in a rich hydrogen bonding network. Due to these hydrogen bonds between coordinated water molecules and coordinated/uncoordinated carboxylate oxygen atoms, one-dimensional chains are alternatively packed into a three-dimensional network. The lengths of these H_2_O…O interactions correspond to the average strength of H-bonds (2.636(6)–2.844(6) Å). The coordinated water molecules in the ChelZn structure do not exhibit significant ππ stacking interactions. In the zinc chelidonate molecule, the ligand binds to the metal cation, forming a chelate complex. Similar structures of zinc complexes with chelidonic acid have been described in the literature. The complex obtained in our study differs in composition and structure. In the work zinc complexes with chelidonic acid in methanol were synthesized [32]. The resulting compound was then recrystallized in DMSO. The resulting complex had the following composition: ZnChel(DMSO)_2_. In this compound, the coordination number of zinc is 4. The inner coordination sphere contains two oxygen atoms from two carboxylate ions of chelidonic acid and two oxygen atoms from two DMSO molecules. In the work [33] a zinc complex with chelidonic acid of the following composition Zn(Chel)_2_(H_2_O)_2_ was obtained by hydrothermal synthesis. This structure also represents a coordination polymer in which the zinc cation has a coordination number of 4. In the first coordination sphere of this complex there are two oxygen atoms from the carboxylate ions of chelidonic acid and two oxygen atoms from two water molecules.

The carboxylate groups in the chelidonate ligand exhibit asymmetry in their C–O bond lengths. For example, in one carboxylate unit (C2), the C–O bond involving the coordinated oxygen (O3, bound to Zn) is C2–O3 = 1.206(12) Å, while the bond to the uncoordinated oxygen is C2–O2 = 1.212(16) Å (see Table S3 for full details and symmetric equivalent in the other carboxylate group at C1: C1–O1 = 1.232(16) Å and C1–O6 = 1.196(13) Å). Although the difference (Δ ≈ 0.006–0.036 Å) is small and within experimental error margins, this asymmetry is indicative of monodentate (or bridging) coordination mode, where the electronic distribution is perturbed by metal binding, leading to non-equivalent C–O bonds. In contrast, symmetric bidentate chelation would typically show more equivalent C–O lengths (both ≈1.25 Å with minimal Δ). This observation aligns with the monodentate/bridging mode observed in the X-ray structure and is consistent with examples in ref. [35], where similar bond length differences confirm monodentate carboxylate coordination in related metal complexes.

#### 2.1.7. Behavior in Aqueous Solution

To evaluate the efficiency of interactions between chelidonic acid anions and Zn^2+^ cations in aqueous solution, a molecular dynamics simulation was performed using the GFN2-XTB semiempirical quantum chemical scheme. The initial and final molecular assemblies are shown in Figure S10.

It was previously shown [31] that in the case of magnesium chelidonate, the formation of a hexahydrate shell of Mg^2+^ cations is preferable, with the chelidonate anion located in the outer sphere and bound to the water molecules of the magnesium hydration shell via a system of hydrogen bonds. However, in the process of simulating an aqueous solution of zinc chelidonate, it was found that under the specified conditions, monodentate coordination of the chelidonate anion with the zinc cation is more preferable, resulting in the formation of a complex of the composition ZnChel(H_2_O)_5_.

**Figure 4:**
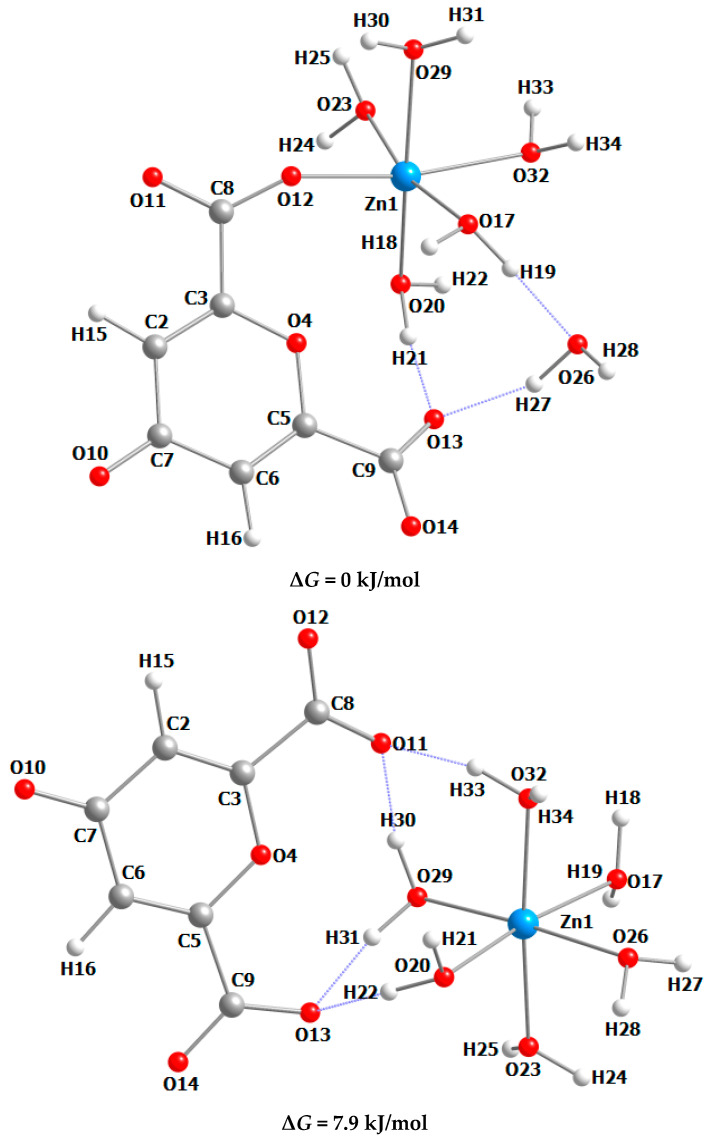
Optimized structures and relative energies of molecular associates of ZnChel(H_2_O)_5_ H_2_O and [Zn(H_2_O)_6_]^2+^ Chel^2−^ compositions (calculation at the ωB97X-3c level).

To more accurately assess the stability of the simulated systems, a comparative quantum chemical energy calculation was performed for the ZnChel(H_2_O)_5_ H_2_O system with direct Zn-Chel coordination and the [Zn(H_2_O)_6_]^2+^ Chel^2−^ system, in which the chelidonate anion is located in the outer sphere and is bound to water molecules of the zinc hydration shell via a system of hydrogen bonds. The optimized structures and relative energies of the studied systems are shown in Figure 4. The calculated geometric parameters of the optimized structures are presented in Table S6. As can be seen, in both cases the Zn^2+^ cation has a distorted octahedral environment, with the Zn-O bond with the oxygen atom of the carboxylate group in ZnChel(H_2_O)_5_ H_2_O being shorter than similar bonds with the oxygen atoms of water molecules. According to calculations, the system with direct monodentate Zn-Chel coordination is thermodynamically more stable, indicating a low probability of the existence of hexaaqua cations [Zn(H_2_O)_6_]^2+^ in aqueous solution in the presence of excess chelidonate anions.

Thus, the quantum-chemical modeling data are consistent with the X-ray diffraction data for zinc chelidonate in the crystalline state, which indicates a clear preference for monodentate coordination of chelidonate anions with zinc cations. In this case, a complex compound with bridging coordination of zinc tetraaqua cations by chelidonate anions is released from the solution, leading to the formation of a coordination polymer of the composition [ZnChel(H_2_O)_4_]n.

### 2.2. Interaction of Zinc Chelidonate and the BSA Molecule

Serum albumins are widely used as model proteins in studies of metal–protein interactions and in the development of metal-based therapeutic agents [38]. In this work, bovine serum albumin was selected as a model system because it is structurally well characterized and commonly used in ligand-binding studies [39]. The presence of two tryptophan residues makes BSA particularly suitable for fluorescence-based analysis.

#### 2.2.1. Fluorescence Quenching Spectra

**Figure 5:**
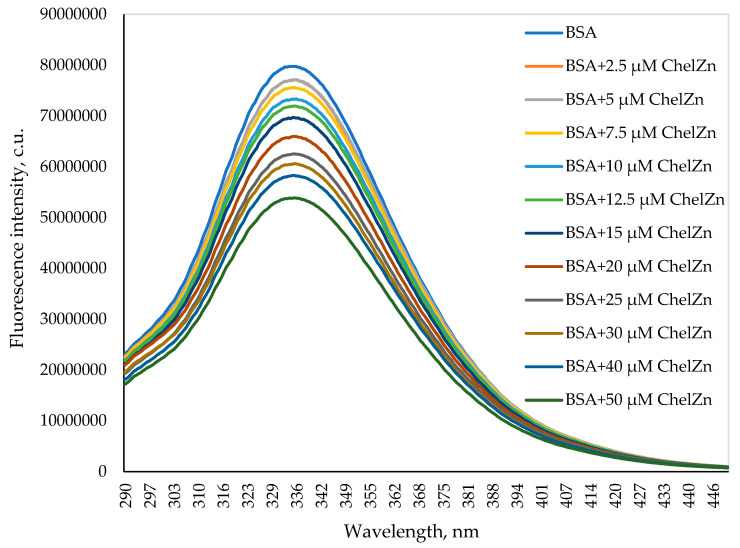
Intrinsic fluorescence spectra (*λex* = 280 nm) of BSA (5 μM) in the absence and presence of zinc chelidonate (ChelZn).

Figure 5 shows the intrinsic fluorescence spectra of BSA (5 μM) in a buffer solution in the absence and presence of increasing concentrations of zinc chelidonate (ChelZn), recorded at an excitation wavelength of 280 nm. BSA exhibits a fluorescence emission maximum at 336 nm, arising predominantly from tryptophan residues. The increase in ChelZn results in a concentration-dependent decrease in fluorescence intensity, indicating quenching of BSA intrinsic fluorescence.

#### 2.2.2. Mechanisms of Quenching of Intrinsic Fluorescence

Fluorescence quenching experiments were conducted at three temperatures (297, 303, and 309 K). The quenching mechanism was evaluated using the Stern–Volmer equation:

$$\frac{F_{0}}{F}=1+K_{sv}\times \left[Q\right]=1+K_{q}\times \tau_{0}\times \left[Q\right]$$

Here:

*F*_0_—fluorescence intensity of BSA; *F*—fluorescence intensity in the presence of quencher; *K_sv_—*Stern–Volmer quenching constant; [*Q*]—quencher concentration; *Kq*—bimolecular quenching rate constant; *τ*_0_*—*average excited-state lifetime of the fluorophore in the native protein (10^−8^ s).

**Figure 6:**
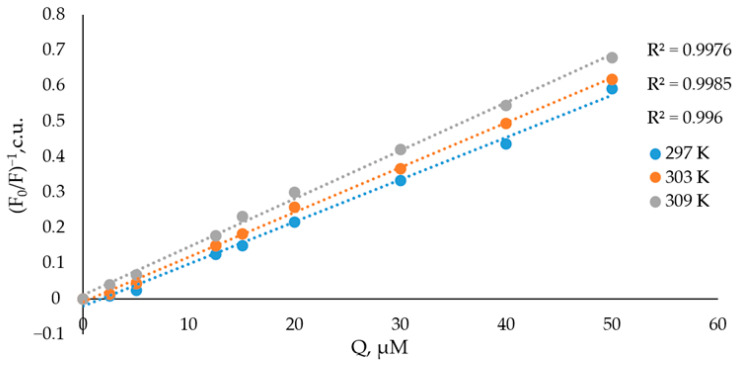
Stern–Volmer graphs plotted in different temperature conditions.

Stern–Volmer plots of (*F*_0_*/F*)^−1^ versus [*Q*] were constructed at each temperature, and the corresponding values of *Ksv* and *Kq* were determined from the slopes (Figure 6) [40].

Fluorescence quenching generally occurs via two mechanisms, dynamic (collisional) and static (complex formation), which can be distinguished by their temperature dependence. In dynamic quenching, the Stern–Volmer constant (*Ksv*) increases with temperature due to enhanced diffusional collisions. In contrast, static quenching is typically characterized by decreasing *Ksv* at higher temperatures, reflecting reduced stability of the ground-state fluorophore–quencher complex. In the present study, *Ksv* increases with temperature, indicating that dynamic quenching contributes significantly to the observed fluorescence decrease, consistent with the increased frequency of collisions between BSA and zinc chelidonate. However, the calculated bimolecular quenching rate constants (*Kq*) exceed the diffusion-controlled limit for biological macromolecules (~2.0 × 10^10^ M^−1^s^−1^), which is typically considered diagnostic of static quenching [41,42]. These results therefore suggest that both collisional and complex-formation processes contribute to the overall quenching mechanism. In this study, the bimolecular quenching rate constants were calculated as 1.191(±0.021) × 10^12^, 1.263 (±0.028) × 10^12^, and 1.366 (±0.034) × 10^12^ M^−1^s^−1^, at 297, 303, and 309 K, respectively. Since these values substantially exceed the diffusion-controlled limit. They are consistent with the presence of static interaction between BSA and zinc chelidonate (Table 2).

Thus, quenching of the intrinsic tryptophan and tyrosine fluorescence of BSA by zinc chelidonate proceeds via a mixed static–dynamic mechanism, consistent with observations reported for other protein–ligand systems [42].

The binding constant (*K_a_*) and the number of binding sites (*n*) were determined using a modified Scatchard equation [43].

$$\log\frac{\left(F_{0}-F\right)}{F}=\log K_{a}+n\times \log\left[Q\right]$$

**Figure 7:**
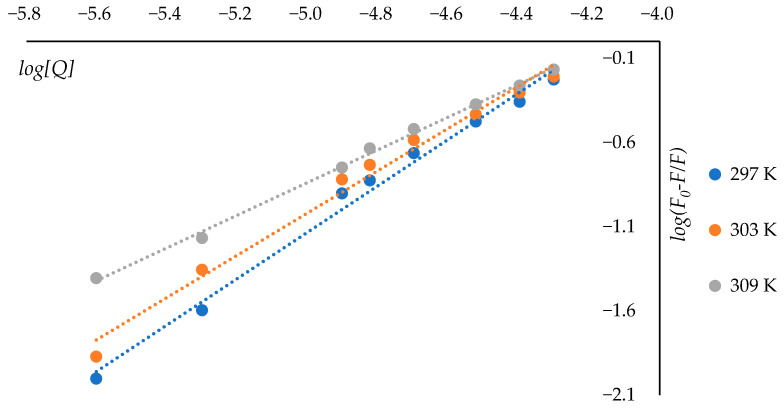
Fluorescence quenching graphs at different temperature conditions, plotted in Scatter coordinates.

Figure 7 shows the graphs in coordinates $\log\frac{\left(F_{0}-F\right)}{F}$ oт $\log\left[Q\right]$ at different temperatures. From the linear regression of these plots, the binding constant (*K_a_*) and the number of binding sites (*n*) are obtained according to the modified Scatchard approach [43]. The calculated values are summarized in Table 2. A decrease in *K_a_* with increasing temperature was observed, further supporting the contribution of dynamic interactions to the protein–ligand binding process.

To elucidate the nature of intermolecular interactions between zinc chelidonate and BSA, thermodynamic parameters were determined using the van’t Hoff approach [44]. The Gibbs free energy (Δ*G*) of binding was calculated from the binding constant *Ka* using:

$$\Delta G=-RT\ln K_{a}$$

and the Gibbs-Helmholtz equation [44]:

$$\Delta G=\Delta H-T\Delta S=-RT\ln K_{a}$$

$$-\frac{\Delta H}{RT}+\frac{\Delta S}{R}=\ln K_{a}$$

**Figure 8:**
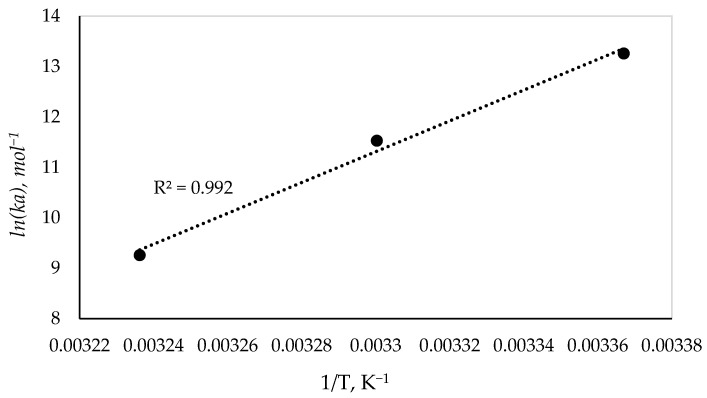
Dependence of *ln*(*Ka*) on 1/T for the BSA-ChelZn complex.

Enthalpy (Δ*H*) and entropy (Δ*S*) contributions were subsequently evaluated. The values of ∆*H* and ∆*S* were obtained from the slope and intercept of the van’t Hoff plot of ln(*K_a_*) versus 1/T (Figure 8) [45].

The change in the Gibbs free energy (∆*G*) at 297 K was calculated as—31.14 kJ mol^−1^, indicating that formation of the BSA–ChelZn complex is a spontaneous process. The enthalpy and entropy changes were determined as −208.94 kJ mol^−1^ and −598.00 J mol^−1^ K^−1^, respectively. Because both ∆*H* < 0 and ∆*S* < 0, the binding interaction is dominated by hydrogen bonding and van der Waals forces, consistent with literature reports for protein–ligand systems governed by specific, non-covalent interactions [46,47].

#### 2.2.3. Synchronous Fluorescence

**Figure 9:**
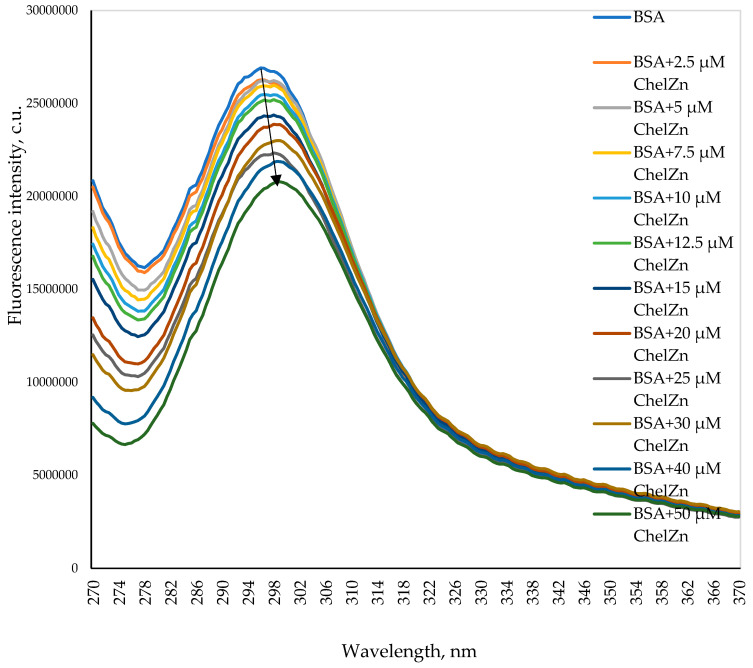
Synchronous fluorescence spectra of BSA at ∆λ = 15 nm in the absence and presence of zinc chelidonate (ChelZn).

Synchronous fluorescence spectroscopy enables selective analysis of tyrosine and tryptophan residues when the wavelength interval between excitation and emission is set to 15 nm and 60 nm, respectively [42]. Figure 9 shows the synchronous fluorescence spectra of BSA at ∆*λ* = 15 nm in the absence and presence of increasing concentrations of ChelZn. These spectra reflect changes in the local environment of tyrosine residues. Progressive addition of ChelZn results in a concentration-dependent decrease in fluorescence intensity accompanied by a 2–3 nm red shift in the emission maximum. This bathochromic shift indicates an increase in the polarity of the microenvironment surrounding tyrosine residues in the BSA molecule.

The increase in polarity may be due to the formation of new hydrogen bonds with the polar groups of chelidonic acid residues in the ChelZn complex. Another possible scenario is an increase in the number of contacts of tyrosine residues with solvent molecules against the background of conformational changes due to the interaction of the protein with ChelZn [42].

**Figure 10:**
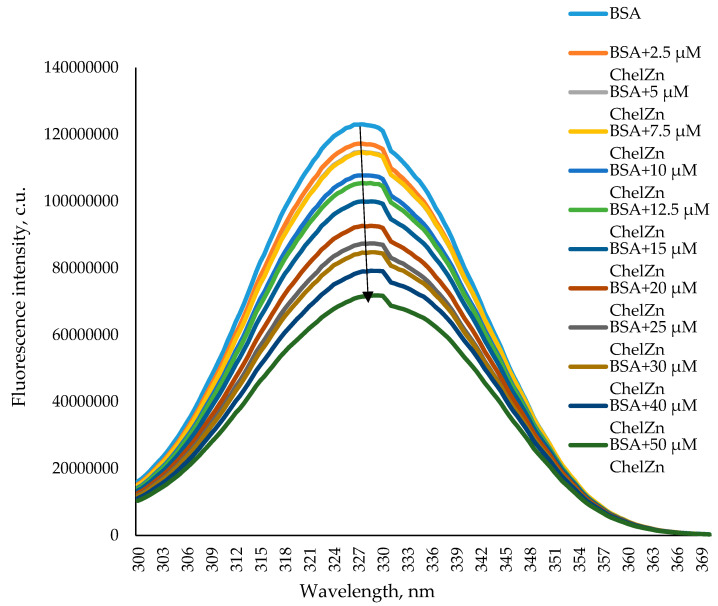
Synchronous fluorescence spectra (∆λ = 60 nm) of bovine serum albumin (BSA) at a concentration of 5 µM in the absence and with the addition of zinc chelidonate (ChelZn).

Figure 10 presents the synchronous fluorescence spectra of BSA at *∆λ* = 60 nm in the absence and presence of ChelZn in solution. It reflects the microenvironment of tryptophan residues. Increasing concentrations of ChelZn produce a concentration-dependent decrease in fluorescence intensity, accompanied by a small red shift of approximately 1 nm. This modest bathochromic shift suggests only minor changes in the polarity surrounding tryptophan residues within the BSA structure. Based on the experimental data obtained on synchronous fluorescence spectra, the following conclusion can be drawn when ChelZn interacts with the BSA molecules: the environment of tyrosine amino acid residues undergoes the greatest changes.

#### 2.2.4. Absorption Spectra

**Figure 11:**
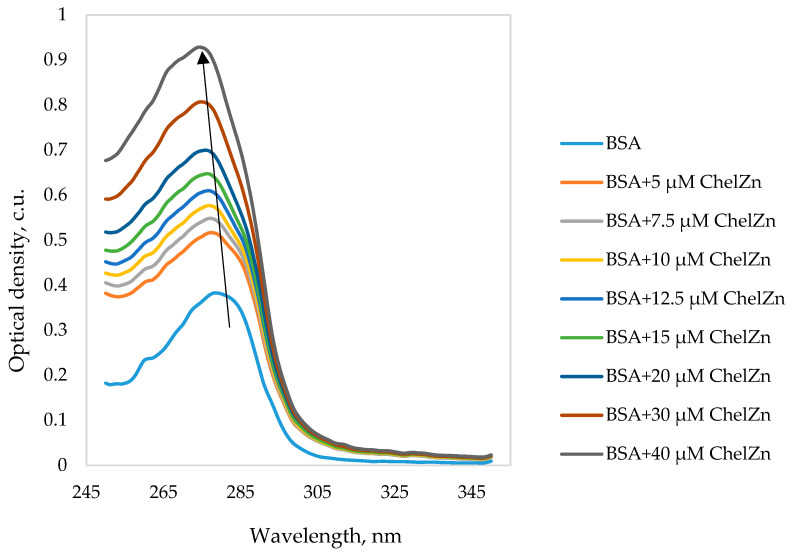
Absorption spectra of bovine serum albumin (BSA) at a concentration of 5 µM in the absence and with the addition of zinc chelidonate (ChelZn).

Figure 11 shows the UV–Vis absorption spectra of BSA in the absence and presence of ChelZn.

With increasing concentrations of ChelZn, the UV–Vis spectra of the BSA–ChelZn system show an increase in absorbance accompanied by a hypsochromic shift of approximately 5 nm. The increase in optical density may arise from two contributing factors. First, enhanced light scattering may occur due to changes in the hydrodynamic dimensions of the BSA globule upon interaction with ChelZn. Second, true absorption may increase because of conformational rearrangements in the vicinity of aromatic amino acid residues. The accompanying blue shift in the absorption maximum supports the latter mechanism, reflecting changes in the polarity of the microenvironment surrounding tryptophan and tyrosine chromophores. Migration of these residues from a relatively hydrophobic protein interior to a more polar aqueous environment stabilizes the ground electronic state, increasing the energy required for excitation and shifting the absorption band to shorter wavelengths.

**Figure 12:**
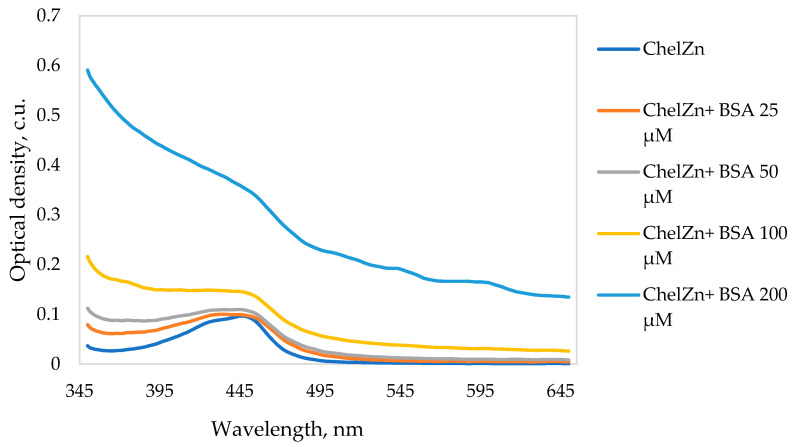
Absorption spectra of zinc chelidonate (ChelZn) 1 mM in the absence and with the addition of bovine serum albumin (BSA) at various concentrations.

The absorption behavior of ChelZn alone was also examined. The complex exhibits a characteristic visible absorption maximum at 447 nm (Figure 12), which can serve as a diagnostic feature for monitoring ligand–protein interactions under appropriate concentration conditions. This maximum absorption is caused by a π → π* transition in the ligand molecule. Hyperchromism upon interaction with BSA may indicate a change in the ligand environment [48]. When the ligand interacts with the protein molecule, the dielectric permittivity of its microenvironment changes, and the ligand becomes embedded in a less polar environment. As a result, the probability of the π → π* transition in the ligand molecule increases. Since the complex was incubated with BSA for 24 h, the optical density of ChelZn was measured at 447 nm at baseline and 24 h later. No differences in optical density values were detected. This indicates stability of the complex in solution during incubation.

#### 2.2.5. Circular Dichroism Spectra

**Figure 13:**
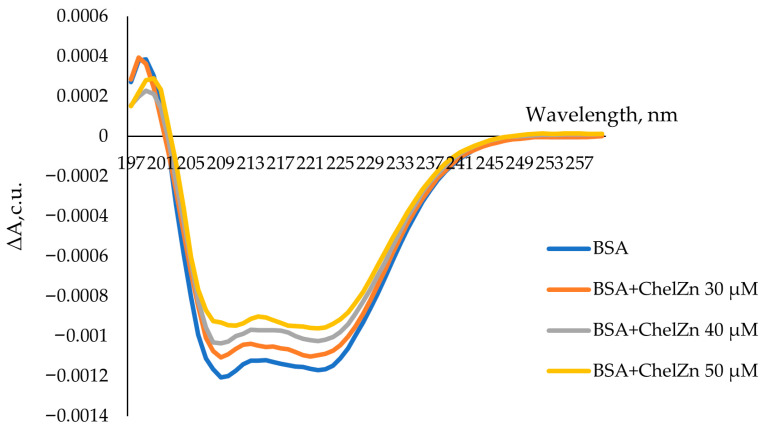
CD spectra of bovine serum albumin (BSA, 5 μM) in the absence and presence of ChelZn.

Figure 13 shows the CD spectra of BSA in the absence and presence of ChelZn. In both cases, the spectra exhibit two negative bands near 208 and 220 nm, characteristic of α-helical secondary structure. Upon addition of ChelZn, the overall CD signal intensity decreases, reflecting a reduction in the difference between left- and right-circularly polarized light absorption (Δε).

The α-helical content of BSA is primarily determined by the ellipticity at 208 and 220 nm. The observed decrease in Δ*ε* at these wavelengths indicates a reduction in *α*-helix content, implying that ChelZn induces partial perturbation of the secondary structure. In the native structure of BSA, the α-helix content was 47.1%, which is consistent with the literature data [49]. Upon interaction with zinc chelidonate at a concentration of 30 µM, the α-helical content decreased to 40.8%. At ChelZn concentrations of 40 and 50 µM, the α-helix content further decreased to 38% (Figure S11).

This structural modification may arise from disruption of hydrogen bonding along the peptide backbone and local unwinding of the α-helical domains upon ligand binding [49].

Based on the optical studies performed, the following conclusions can be drawn. The experiments on quenching of the intrinsic fluorescence of BSA by zinc chelidonate molecules showed that the process proceeds via a mixed mechanism. On the one hand, dynamic quenching is observed, caused by collisional encounters between the fluorophore and quencher molecules. Dynamic quenching is indicated by the direct proportionality between the Stern–Volmer constant and temperature. Further spectroscopic investigations (synchronous fluorescence, atomic absorption, and CD spectroscopy) point to changes in the secondary and tertiary structure of BSA induced by direct interaction with the ligand. On this basis, we assume that static quenching provides the main contribution to fluorescence quenching. As the temperature increases, the protein structure becomes less accessible to the complex, and the contribution of the dynamic quenching mechanism increases. Similar behavior has been observed for other metal complexes in their interactions with serum albumins [50]. The calculated stability constant for the zinc chelidonate–BSA complex is of the same order of magnitude as the stability constants reported for other metal complexes with serum albumins, for which a static mechanism of intrinsic protein fluorescence quenching has likewise been established [51,52,53]. Based on the experimental data obtained in the BSA model system, it demonstrates that zinc chelidonate interacts with serum albumin and induces measurable conformational changes. These findings suggest that albumin may act as a carrier protein for zinc chelidonate following systemic administration in vivo. It should be noted that the synthesis of substances with biological activity is an important scientific direction with great prospects for the implementation of the obtained results [54,55].

## 3. Materials and Methods

### 3.1. Materials

Chelidonic acid (4-oxo-4H-pyran-2,6-dicarboxylic acid) and zinc oxide (Macklin, Shanghai, China) were used as received without further purification.

- Chelidonic acid (H_2_Chel):

IR (ATR), ν (cm^−1^): 3524, 3371 (O–H); 3092 (C–H); 1750 (C=O); 1675 (COOH); 1623, 1577, 1504, 1392, 1269, 1232, 1195, 1046, 997, 919, 906, 781, 757, 669, 546, 498.

^1^H NMR (D_2_O, δ ppm): 7.11 (s, 2H, H-3, H-5; ^1^JH-C = 172.2 Hz, ^3^JH-H = 2.20 Hz).

^13^C NMR (D_2_O, δ ppm): 117.4 (C-3, C-5); 157.7 (C-2, C-6); 163.9 (COOH); 184.4 (C=O).

Absorption spectrum, nm: 225 (ε = 6800 dm^3^ × M^−1^ × cm^−1^; C = O, π → π*); 271 (ε = 11,200 dm^3^ × M^−1^ × cm^−1^; COO^−^, π → π*).

- Zinc chelidonate [Zn(C_7_H_2_O_6_)(H_2_O)_4_]_n_

**Figure 14:**
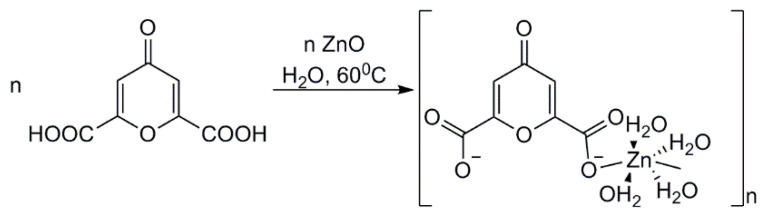
Preparation of zinc chelidonate.

Zinc chelidonate complex [Zn(Chel)(H_2_O)_4_]n was synthesized by the reaction between chelidonic acid and zinc oxide in an aqueous medium at 60 ± 2 °C. (Figure 14). Chelidonic acid (0.45 g, 2.4 mmol) was dissolved in 15 mL of water at 60 ± 2 °C. Zinc oxide (0.26 g, 1.2 mmol) was added portionwise with stirring at constant temperature. The solution became yellow (pH 5.5–6.0). The reaction mixture was stirred for 5–6 h. Crystallization occurred upon evaporation to approximately 75% of the original volume. The product was isolated and recrystallized from bidistilled water to give pale crystals in 80% yield (0.307 g, 0.96 mmol). The final product was isolated by filtration, washed with cold bidistilled water, and dried in a desiccator over silica gel under reduced pressure (high vacuum pump) for 24 h to minimize adsorbed moisture.

- IR (ATR), ν (cm^−1^): 3103 (br), 1720 (sh), 1619, 1582 (νas COO^−^), 1431, 1355 (νs COO^−^), 1122, 964, 932, 915, 822, 800, 747, 695, 537, 511, 496.
- ^1^H NMR (D_2_O, δ ppm): 7.01 (s, 2H, H-3, H-5; ^1^JH-C = 171.9 Hz, ^3^JH-H = 2.34 Hz).
- ^13^C NMR (D_2_O, δ ppm): 116.3 (C-3, C-5); 159.6 (C-2, C-6); 165.2 (COO^−^); 185.1 (C=O).

Absorption spectrum, nm: 225 (ε = 23,680 dm^3^ × M^−1^ × cm^−1^; C=O, π → π*); 272 (ε = 14,000 dm^3^ × M^−1^ × cm^−1^; COO^−^, π → π*); 447 (ε = 110 dm^3^ × M^−1^ × cm^−1^, Zn-O, n→ π*).

Found (%): C 26.23; H 3.23; O 49.48; Zn 20.62.

Calculated for ZnChel(H_2_O)_4_ (%): C 26.29; H 3.12; O 50.10; Zn 20.34.

### 3.2. Measurements

Single-crystal X-ray diffraction data were collected on an Agilent SuperNova dual-source diffractometer equipped with a Cu Kα microfocus source and an AtlasS2 CCD detector (Agilent Technologies Singapore International, Singapore, Singapore) using Cu Kα radiation (λ = 1.54178 Å) at 100 K. Absorption correction was applied using SADABS. The structure was solved by direct methods and refined by full-matrix least squares on F^2^ using SHELXTL, with anisotropic refinement for all non-hydrogen atoms. Hydrogen atoms were located from different Fourier maps and refined isotopically.

The complex crystallizes in the triclinic space group P-1 with the following crystallographic parameters:

F_w_ = 319.52 g·mol^−1^;

a = 5.022(3) Å; b = 5.2447(14) Å; c = 9.9070(7) Å;

*α* = 94.470°; *β* = 93.965(6) °; *γ* = 98.497(6)°;

V = 256.44(3) Å^3^; Z = 1;

μ(CuK_α_) = 3.886 mm^−1^;

Dcalc = 2.069 g·cm^−3^;

F(000) = 162.0.

Data were collected over the range 8.94 ≤ 2θ ≤ 151.76°.

Reflections measured: 5279; independent reflections: 5279 (R_int_ = 0.0078).

Refined parameters: 132.

Final refinement statistics:

R_1_ = 0.0619, wR_2_ = 0.1168 for reflections with I > 2σ(I);

R_1_ = 0.0620, wR_2_ = 0.1679 for all data;

Goodness-of-fit on F^2^ = 1.073;

Residual electron density: Δρ_max_/Δρ_min_ = 1.72/−1.01 e·Å^−3^.

Crystallographic data have been deposited with the Cambridge Crystallographic Data Centre (CCDC 2470180).

IR spectra were recorded on a VERTEX 70 FTIR spectrometer (Bruker, Ettlingen, Germany) over the range 4000–400 cm^−1^ using an ATR accessory. Data processing was performed using the instrument software and SpecMan (ACDLabs 10.0).

^1^H and ^13^C NMR spectra were obtained on a JNM-ECA 400 spectrometer (JEOL, Tokyo, Japan) at 400 and 101 MHz, respectively, in D_2_O at 298 K. Residual solvent peaks were used as internal standards. Data were processed using NMRMan (ACDLabs 10.0).

Thermogravimetric analysis (TGA) was carried out on an STA-409 PC Luxx thermal analyzer (Netzsch, Selb, Germany) in air using alumina crucibles and an α-Al_2_O_3_ reference. Samples were heated from 30 to 1000 °C at 10 °C·min^−1^.

Elemental composition was determined by X-ray fluorescence using a Shimadzu EDX-8000 analyzer (Shimadzu, Kyoto, Japan). Classical CHN analysis was performed on a EuroVector EA3000 instrument (Eurovector S.p.A., Pavia, Italy).

Circular dichroism (CD) spectra were recorded on a Mark V dichrograph (Jobin Yvon, Longjumeau, France) at room temperature in 0.1-cm quartz cuvettes. Spectra were acquired from 197 to 260 nm with 1 nm resolution. Each point represented the mean of 1000 scans, and three replicate spectra were averaged to minimize stochastic noise.

### 3.3. Computational Methods

Quantum-chemical calculations were carried out using the ORCA 6.0.1 software package, and molecular structures were visualized with ChemCraft 1.8. Molecular dynamics (MD) simulations were performed employing the GFN2-XTB semiempirical method under NHC thermostat control at 353 K with a 1 fs timestep [56].

To refine molecular geometries and evaluate thermodynamic stability, geometry optimizations were conducted using the ωB97X-3c new hybrid method [57], which combines the ωB97X functional with the vDZP basis set and the D4 dispersion correction [58]. The choice of this method was determined by its sufficiently high accuracy in describing thermochemistry and noncovalent interactions, comparable to that of standard hybrid DFT methods employing quadruple-zeta (QZ) basis sets, while requiring significantly lower computational cost [57,59]. Relative Gibbs free energies in aqueous solution were obtained within the CPCM continuum solvation model [60].

In simulations involving direct coordination of the chelidonate anion to the Zn^2+^ cation, additional water molecules were added using the built-in solvator procedure within the GFN2-XTB framework. The resulting systems were subsequently optimized and re-evaluated at the ωB97X-3c level.

### 3.4. Binding Studies with BSA

A commercial lyophilized preparation of bovine serum albumin (BSA) (Sigma Aldrich, Kankakee, IL, USA) was used in the experiment. The interaction of zinc chelidonate (ChelZn) with BSA was studied using optical methods. The ligand–protein interaction was carried out in a medium prepared on a Tris-HCl buffer with pH 7.45 and a concentration of 0.01 M. This buffer was chosen because of its minimal interaction with the solution components. All solutions were prepared using water obtained from a Mili-Q Advantage system. Immediately before measurement, the BSA and ligand solutions were incubated in a thermostat for 24 h at 297 K. This incubation time was chosen to achieve maximal binding of the ligand to the protein. All measurements were also performed in thermostatted cells at 297 K. In the experiments on the temperature dependence of BSA fluorescence, the BSA and ligand solutions were likewise incubated for 24 h in thermostats at additional temperatures of 300 and 309 K. The measurements were correspondingly carried out at these temperature conditions. All solutions were sterilized prior to incubation using sterile filtration units with a pore size of 0.45 µm. All optical measurements were performed in three independent experiments.

Fluorescence measurements were obtained using a Shimadzu RF-6000 spectrofluorimeter (Shimadzu, Kyoto, Japan). The intrinsic fluorescence spectra of BSA in the absence and presence of zinc chelidonate were recorded in the range from 290 to 500 nm at an excitation wavelength of 280 nm. In these experiments, the final concentration of BSA in solution was 5 µM, and the final concentration of zinc chelidonate ranged from 2.5 to 50 µM. All fluorescence spectra are presented with correction for inner filter absorption.

$$F_{corr}=F_{obs}\times 10^{\frac{D_{ex+D_{em}}}{2}}$$

F_corr_—fluorescence intensity after inner-filter correction; F_obs_—measured fluorescence intensity; D_ex_—optical density of the ligand at the excitation wavelength; D_em_—optical density of the ligand at the emission wavelength.

UV–Vis absorption spectra were recorded on a U-2900 double-beam spectrophotometer (Hitachi High-Tech, Tokyo, Japan) using 10-mm quartz cuvettes over the range 190–600 nm. Spectra were smoothed using FFT filtering in OriginLab 2019. Two series of solutions were used to study the absorption spectra of the BSA-ChelZn complex. In the first series, BSA was kept at a constant final concentration of 5 µM, while the concentration of zinc chelidonate was varied from 5 to 40 µM. In this measurement, the presence of interaction was inferred from changes in the protein absorption band at 280 nm. In the second series of experiments, the concentration of zinc chelidonate was fixed at 1 mM, and BSA was added in concentrations ranging from 25 to 200 µM. The interaction between the protein and the ligand was assessed at λ = 447 nm, since this absorption maximum is characteristic of an aqueous solution of zinc chelidonate.

Circular dichroism (CD) spectra were recorded on a Mark V dichrograph (Jobin Yvon, France) at room temperature in 0.1 cm quartz cuvettes. Each point represented the mean of 1000 scans, and three replicate spectra were averaged to minimize stochastic noise. Circular dichroism spectra of BSA in the absence and presence of zinc chelidonate were recorded from 190 to 260 nm with a step size of 1 nm. The final protein concentration in solution was 5 µM, while maximum concentrations of 30, 40, and 50 µM were selected for zinc chelidonate. The CD spectra was analyzed using the BeStSel (https://bestsel.elte.hu/index.php accessed on 10 September 2025) to estimate the percentage of α-helical structure. The signal intensity is expressed in Δε, M^−1^cm^−1^.

## 4. Conclusions

In this study, a zinc complex of chelidonic acid was synthesized by reacting 4-oxo-4H-pyran-2,6-dicarboxylic acid with zinc oxide under isothermal conditions. Elemental and thermogravimetric analyses established the empirical formula C_7_H_10_O_10_Zn, while NMR and IR spectroscopy confirmed the molecular structure. Single-crystal X-ray diffraction revealed a coordination polymer of composition ZnChel(H_2_O)_4_, in which Zn(II) adopts a six-coordinate, distorted octahedral geometry formed by two chelidonate ligands and four water molecules. This coordination mode differs fundamentally from previously reported Zn–chelidonate complexes, which display tetra-coordination.

Quantum-chemical calculations indicated that, in aqueous solution, the dominant species is ZnChel(H_2_O)_5_, characterized by preferential monodentate coordination of the chelidonate anion. The interaction between zinc chelidonate and bovine serum albumin was investigated using fluorescence, UV–Vis absorption, and circular dichroism spectroscopy. The results demonstrate that complex formation is driven primarily by hydrogen bonding and van der Waals forces and is accompanied by measurable changes in the secondary and tertiary structures of BSA.

In conclusion, this study expands current understanding of the coordination chemistry of chelidonic acid and provides the first evidence for protein binding of zinc chelidonate, supporting future biomedical evaluation in vitro and in vivo.

## Figures and Tables

**Table 1:** Comparison of physicochemical analysis data to establish the gross formula of zinc chelidonate [Zn(Chel)(H_2_O)_4_]_n_.

	Found, wt. %			RSA Data
	Elemental Energy Dispersive Analysis	Classical C, H, N, O Analysis	Atomic Absorption	
Zn	21.14 ± 1.2	-	20.1 ± 0.5	20.34
H	3.36 ± 0.5	3.1 ± 0.9	-	3.12
C	26.22 ± 1.1	26.25 ± 0.7	-	26.29
O	49.88 ± 0.7	49.1 ± 1.3	-	50.1

**Table 2:** Main parameters characterizing the interaction of BSA with zinc chelidonate.

Temperature, K	*Ksv* × 10^4^, M^−1^	*Kq* × 10^12^, M^−1^ × s^−1^	*R* ^2^	*n*	*Ka* × 10^3^, M^−1^
298	1.191 ± 0.021	1.191 ± 0.021	0.9976	1.37	571.6102
303	1.263 ± 0.028	1.263 ± 0.028	0.9985	1.25	181.5098
309	1.366 ± 0.034	1.366 ± 0.034	0.9962	0.97	10.47611

## Data Availability

The full dataset is available in the Supplementary Material.

## Supplementary Materials

The following supporting information can be downloaded at: https://www.mdpi.com/article/10.3390/molecules31091378/s1, Figure S1: ^1^H NMR spectrum (400 MHz, D_2_O) of chelidonic acid. Figure S2: Spin-spin interaction constants in the minor isotopomer of chelidonic acid. Figure S3: ^13^C NMR spectrum (101 MHz, D_2_O) of chelidonic acid. Figure S4: Assignment of signals in the ^13^C NMR spectrum of chelidonic acid. Figure S5: ^1^H NMR spectrum (400 MHz, D_2_O) of zinc chelidonate [Zn(Chel)(H_2_O)_4_]_n_. Figure S6: Spin-spin interaction constants in the minor isotopomer of the chelidonate ion. Figure S7: ^13^C NMR spectrum (101 MHz, D_2_O) of zinc chelidonate [Zn(Chel)(H_2_O)_4_]_n_. Figure S8: IR spectra of (**a**) chelidonic acid (**b**) zinc chelidonate [Zn(Chel)(H_2_O)_4_]_n_. Figure S9: Thermal analysis data for zinc chelidonate. Figure S10: Initial and final results of molecular dynamics simulation (calculation at the GFN2-XTB level). Figure S11: CD spectra in the presence and absence of zinc chelidonate. Table S1: Composition of the zinc chelidonate complex according to the results of thermal analysis. Table S2: Crystallographic data, experimental parameters and refinements of the structure of zinc chelidonate. Table S3: Crystallographic distances between atoms. Table S4: Crystallographic angles between atoms. Table S5: Hydrogen bond parameters. Table S6: Geometric parameters of zinc coordination nodes in molecular associates of ZnChel(H_2_O)_5_·H_2_O и [Zn(H_2_O)_6_]^2+^·Chel^2−^ compositions (calculation at the *ω*B97X-3c level).

## Abbreviations

The following abbreviations are used in this manuscript:

NMR	Nuclear magnetic resonance spectroscopy
IR	Infrared spectroscopy
ATR	Attenuated total reflectance
FFT	Fast Fourier transform
BSA	Bovine serum albumin

## References

[1] Kambe T., Wagatsuma T. (2023). Metalation and Activation of Zn^2+^ Enzymes via Early Secretory Pathway-Resident ZNT Proteins. Biophys. Rev..

[2] Amagai Y., Yamada M., Kowada T., Watanabe T., Du Y., Liu R., Naramoto S., Watanabe S., Kyozuka J., Anelli T. (2023). Zinc homeostasis governed by Golgi-resident ZnT family members regulates ERp44-mediated proteostasis at the ER-Golgi interface. Nat. Commun..

[3] Read K.A., Jones D.M., Freud A.G., Oestreich K.J. (2021). Established and emergent roles for Ikaros transcription factors in lymphoid cell development and function. Immunol. Rev..

[4] Wessels I., Maywald M., Rink L. (2017). Zinc as a Gatekeeper of Immune Function. Nutrients.

[5] Haas K.L., Franz K.J. (2009). Application of metal coordination chemistry to explore and manipulate cell biology. Chem. Rev..

[6] Luan R., Ding D., Xue Q., Li H., Wang Y., Yang J. (2023). Protective role of zinc in the pathogenesis of respiratory diseases. Eur. J. Clin. Nutr..

[7] Lee S.R. (2018). Critical Role of Zinc as Either an Antioxidant or a Prooxidant in Cellular Systems. Oxidative Med. Cell. Longev..

[8] Banaszak M., Gorna I., Przysławski J. (2021). Zinc and the Innovative Zinc-α2-Glycoprotein Adipokine Play an Important Role in Lipid Metabolism: A Critical Review. Nutrients.

[9] Ahmad R., Shaju R., Atfi A., Razzaque M.S. (2024). Zinc and Diabetes: A Connection between Micronutrient and Metabolism. Cells.

[10] Harchegani A.B., Dahan H., Tahmasbpour E., Bakhtiari Kaboutaraki H., Shahriary A. (2020). Effects of zinc deficiency on impaired spermatogenesis and male infertility: The role of oxidative stress, inflammation and apoptosis. Hum. Fertil..

[11] Chen B., Yu P., Chan W.N., Xie F., Zhang Y., Liang L., Leung K.T., Lo K.W., Yu J., Tse G.M.K. (2024). Cellular Zinc Metabolism and Zinc Signaling: From Biological Functions to Diseases and Therapeutic Targets. Signal Transduct. Target. Ther..

[12] Zhu D., Su Y., Zheng Y., Fu B., Tang L., Qin Y.-X. (2018). Zinc Regulates Vascular Endothelial Cell Activity through Zinc-Sensing Receptor ZnR/GPR39. Am. J. Physiol. Cell Physiol..

[13] Hara T., Yoshigai E., Ohashi T., Fukada T. (2023). Zinc in Cardiovascular Functions and Diseases: Epidemiology and Molecular Mechanisms for Therapeutic Development. Int. J. Mol. Sci..

[14] Lin P.H., Sermersheim M., Li H., Lee P.H.U., Steinberg S.M., Ma J. (2017). Zinc in Wound Healing Modulation. Nutrients.

[15] Hall A.G., King J.C. (2022). Zinc Fortification: Current Trends and Strategies. Nutrients.

[16] Kim S.J., Kim D.S., Li S., Ahn E.M., Kee J.Y., Hong S.H. (2023). Chelidonic acid ameliorates atopic dermatitis symptoms through suppression the inflammatory mediators in in vivo and in vitro. Biol. Chem..

[17] Singh D.K., Gulati K., Ray A. (2016). Effects of chelidonic acid, a secondary plant metabolite, on mast cell degranulation and adaptive immunity in rats. Int. Immunopharmacol..

[18] Oh H.A., Kim H.M., Jeong H.J. (2011). Beneficial effects of chelidonic acid on a model of allergic rhinitis. Int. Immunopharmacol..

[19] Kim D.S., Kim S.J., Kim M.C., Jeon Y.D., Um J.Y., Hong S.H. (2012). The Therapeutic Effect of Chelidonic Acid on Ulcerative Colitis. Biol. Pharm. Bull..

[20] Avdeeva E., Porokhova E., Khlusov I., Rybalova T., Shults E., Litvinova L., Shupletsova V., Khaziakhmatova O., Sukhodolo I., Belousov M. (2021). Calcium Chelidonate: Semi-Synthesis, Crystallography, and Osteoinductive Activity In Vitro and In Vivo. Pharmaceuticals.

[21] Jeong H.J., Yang S.Y., Kim H.Y., Kim N.R., Jang J.B., Kim H.M. (2016). Chelidonic acid evokes antidepressant-like effect through the up-regulation of BDNF in forced swimming test. Exp. Biol. Med..

[22] Khairnar S.I., Kulkarni Y.A., Singh K. (2025). Neuroprotective Effect of Chelidonic Acid through Oxidative Stress Reduction in Paclitaxel-Induced Peripheral Neuropathy in Rats. Naunyn-Schmiedeberg’s Arch. Pharmacol..

[23] Khairnar S.I., Kulkarni Y.A., Singh K. (2025). Cardioprotective Effect of Chelidonic Acid against Doxorubicin-Induced Cardiac Toxicity in Rats. Rev. Port. Cardiol..

[24] Gureev A.P., Chernyshova E.V., Krutskikh E.P., Sadovnikova I.S., Tekutskaya E.E., Dorohova A.A. (2024). Role of the NRF2/ARE Pathway in the mtDNA Reparation. Front. Biosci. Landmark.

[25] Kozin S.V., Lyasota O.M., Kravtsov A.A., Kozlova E.A., Rubailo A.D., Moiseev A.V., Popov K.A., Bespalov A.V., Gordeev K.V. (2025). Comparative Study of Antioxidant Activity of 4-H Pyran Acids Using Optical Methods and Quantum-Chemical Calculations. Biophysics.

[26] Kozin S., Kravtsov A., Ivashchenko L., Dotsenko V., Vasilyeva L., Vasilyev A., Tekutskaya E., Aksenov N., Baryshev M., Dorohova A. (2023). Study of the Magnesium Comenate Structure, Its Neuroprotective and Stress-Protective Activity. Int. J. Mol. Sci..

[27] Kozin S.V., Ivashchenko L.I., Kravtsov A.A., Vasilyeva L.V., Vasiliev A.M., Bukov N.N., Dorohova A.A., Lyasota O.M., Bespalov A.V., Dzhimak S.S. (2023). Meconic Acid Is a Possible Neuroprotector: Justification Based on in vitro Experiments and Its Physicochemical Properties. Biophysics.

[28] Dotsenko V.V., Varzieva E.A., Buriy D.S., Aksenov N.A., Aksenova I.V. (2022). First Synthesis of 2-Amino-5-hydroxy-4H-chromene-3-carbonitriles from 4-(2-Pyridylazo)resorcinol. Russ. J. Gen. Chem..

[29] Dotsenko V.V., Varzieva E.A. (2022). Synthesis of 6-(aryldiazenyl)-4H-chromene derivatives (microreview). Chem. Heterocycl. Compd..

[30] Yasodha V., Govindarajan S., Low J.N., Glidewell C. (2007). Cationic, neutral and anionic metal(II) complexes derived from 4-oxo-4H-pyran-2,6-dicarboxylic acid (chelidonic acid). Acta Crystallogr. C.

[31] Kozin S.V., Kravtsov A.A., Kindop V.K., Bespalov A.V., Ivaschenko L.I., Nazarenko M.A., Moiseev A.V., Churakov A.V., Vashurin A.S. (2025). Synthesis and Physicochemical Properties of Magnesium Salts of 4H-Pyrancarboxylic Acids. Russ. J. Inorg. Chem..

[32] Lago A.B., Carballo R., Fernández-Hermida N., Rodríguez-Hermida S., Vázquez-López E.M. (2011). Coordination polymers with chelidonate (4-oxo-4H-pyran-2,6-dicarboxylate) anions and dmso: [Zn(chel)(dmso)_2_] and linkage isomers of [Co(chel)(dmso)(OH_2_)_3_]·H_2_O. J. Mol. Struct..

[33] Lin X.-M., Chen L., Fang H.-C., Zhou Z.-Y., Zhou X.-X., Chen J.-Q., Xu A.-W., Cai Y.-P. (2009). Construction of three one-dimensional zinc(II) complexes containing pyrazine-2,3-dicarboxylic acid. Inorganica Chim. Acta.

[34] Hagen R.J.D.R., Roberts J.D. (1969). Nuclear magnetic resonance spectroscopy. Carbon-13 spectra of aliphatic carboxylic acids and carboxylate anions. J. Am. Chem. Soc..

[35] Zhou X.X., Liu M.S., Lin X.M., Fang H.C., Chen J.Q., Yang D.Q., Cai Y.P. (2009). Construction of three low-dimensional Zn(II) complexes based on different organic-carboxylic acids. Inorganica Chim. Acta.

[36] Deacon G.B., Phillips R.J. (1980). Relationships between the carbon-oxygen stretching frequencies of carboxylato complexes and the type of carboxylate coordination. Coord. Chem. Rev..

[37] Sutton C.C.R., da Silva G., Franks G.V. (2015). Modeling the IR Spectra of Aqueous Metal Carboxylate Complexes: Correlation between Bonding Geometry and Stretching Mode Wavenumber Shifts. Chem. Eur. J..

[38] Sciortino G., Maréchal J.-D., Garribba E. (2021). Integrated approaches to the characterization of vanadium-protein and enzyme systems. Inorg. Chem. Front..

[39] Grabowska O., Kogut M.M., Żamojć K., Samsonov S.A., Makowska J., Tesmar A., Chmur K., Wyrzykowski D., Chmurzyński L. (2021). Effect of Tetraphenylborate on Physicochemical Properties of Bovine Serum Albumin. Molecules.

[40] Gao X., Bi H., Jia J., Tang L. (2017). Spectroscopic and in silico study of binding mechanism of cyanidin-3-O-glucoside with human serum albumin and glycated human serum albumin. Luminescence.

[41] Xu J., Wang M., Zheng Y., Tang L. (2019). Spectroscopic Technique-Based Comparative Investigation on the Interaction of Theaflavins with Native and Glycated Human Serum Albumin. Molecules.

[42] Cazacu N., Chilom C.G., David M., Florescu M. (2022). Conformational Changes in the BSA-LT4 Complex Induced by the Presence of Vitamins: Spectroscopic Approach and Molecular Docking. Int. J. Mol. Sci..

[43] Lei S., Xu D., Saeeduddin M., Riaz A., Zeng X. (2017). Characterization of molecular structures of theaflavins and the interactions with bovine serum albumin. J. Food Sci. Technol..

[44] Xu L., Hu Y.X., Li Y.C., Liu Y.F., Zhang L., Ai H.X., Liu H.S. (2017). Study on the interaction of paeoniflorin with human serum albumin (HSA) by spectroscopic and molecular docking techniques. Chem. Cent. J..

[45] Li T., Hu P., Dai T., Li P., Ye X., Chen J., Liu C. (2018). Comparing the binding interaction between β-lactoglobulin and flavonoids with different structure by multi-spectroscopy analysis and molecular docking. Spectrochim. Acta A Mol. Biomol. Spectrosc..

[46] Zhou W., Peng C., Wang D., Li J., Tu Z., Zhang L. (2022). Interaction Mechanism between OVA and Flavonoids with Different Hydroxyl Groups on B-Ring and Effect on Antioxidant Activity. Foods.

[47] Li X., Wang G., Chen D., Lu Y. (2015). β-Carotene and astaxanthin with human and bovine serum albumins. Food Chem..

[48] Singha Roy A., Tripathy D.R., Ghosh A.K., Dasgupta S. (2012). An alternate mode of binding of the polyphenol quercetin with serum albumins when complexed with Cu(II). J. Lumin..

[49] Bao Y., Wang Y., Liu H., Lan J., Li Z., Zong W., Zhao Z. (2025). Co-Existing Nanoplastics Further Exacerbates the Effects of Triclosan on the Physiological Functions of Human Serum Albumin. Life.

[50] Mondal P., Bose A. (2019). Spectroscopic overview of quercetin and its Cu(II) complex interaction with serum albumins. Bioimpacts.

[51] Pantelic L., Skaro Bogojevic S., Andrejević T.P., Pantović B.V., Marković V.R., Ašanin D.P., Milanović Ž., Ilic-Tomic T., Nikodinovic-Runic J., Glišić B.Đ. (2024). Copper(II) and Zinc(II) Complexes with Bacterial Prodigiosin Are Targeting Site III of Bovine Serum Albumin and Acting as DNA Minor Groove Binders. Int. J. Mol. Sci..

[52] Biswas N., Saha S., Khanra S., Sarkar A., Prasad Mandal D., Bhattacharjee S., Chaudhuri A., Chakraborty S., Roy Choudhury C. (2019). Example of two novel thiocyanato bridged copper(II) complexes derived from substituted thiosemicarbazone ligand: Structural elucidation, DNA/albumin binding, biological profile analysis, and molecular docking study. J. Biomol. Struct. Dyn..

[53] Samari F., Hemmateenejad B., Shamsipur M., Rashidi M., Samouei H. (2012). Affinity of two novel five-coordinated anticancer Pt(II) complexes to human and bovine serum albumins: A spectroscopic approach. Inorg. Chem..

[54] Zazeri G., Povinelli A.P.R., Le Duff C.S., Tang B., Cornelio M.L., Jones A.M. (2020). Synthesis and Spectroscopic Analysis of Piperine- and Piperlongumine-Inspired Natural Product Scaffolds and Their Molecular Docking with IL-1β and NF-κB Proteins. Molecules.

[55] Zazeri G., Povinelli A.P.R., Pavan N.M., Jones A.M., Ximenes V.F. (2023). Solvent-Induced Lag Phase during the Formation of Lysozyme Amyloid Fibrils Triggered by Sodium Dodecyl Sulfate: Biophysical Experimental and In Silico Study of Solvent Effects. Molecules.

[56] Bannwarth C., Ehlert S., Grimme S. (2019). GFN2-xTB—An Accurate and Broadly Parametrized Self-Consistent Tight-Binding Quantum Chemical Method with Multipole Electrostatics and Density-Dependent Dispersion Contributions. J. Chem. Theory Comput..

[57] Müller M., Hansen A., Grimme S. (2023). ωB97X-3c: A composite range-separated hybrid DFT method with a molecule-optimized polarized valence double-ζ basis set. J. Chem. Phys..

[58] Grimme S., Antony J., Ehrlich S., Krieg H. (2010). A consistent and accurate ab initio parametrization of density functional dispersion correction (DFT-D) for the 94 elements H-Pu. J. Chem. Phys..

[59] Bursch M., Mewes J.-M., Hansen A., Grimme S. (2022). Best-Practice DFT Protocols for Basic Molecular Computational Chemistry. Angew. Chem..

[60] Tomasi J., Mennucci B., Cammi R. (2005). Quantum Mechanical Continuum Solvation Models. Chem. Rev..

