# Supplementary material for: The Structure of Zinc Chelidonate in the Crystalline Phase, Aqueous Solution and Assessment of the Interaction with Serum Albumin
Source: Molecules. 2026 Apr 22;31(9):1378. doi: 10.3390/molecules31091378 (PMC13164747; doi:10.3390/molecules31091378)
Supplement: Supplementary file 1 [file molecules-31-01378-s001.zip › molecules-4199503-supplementary.pdf]

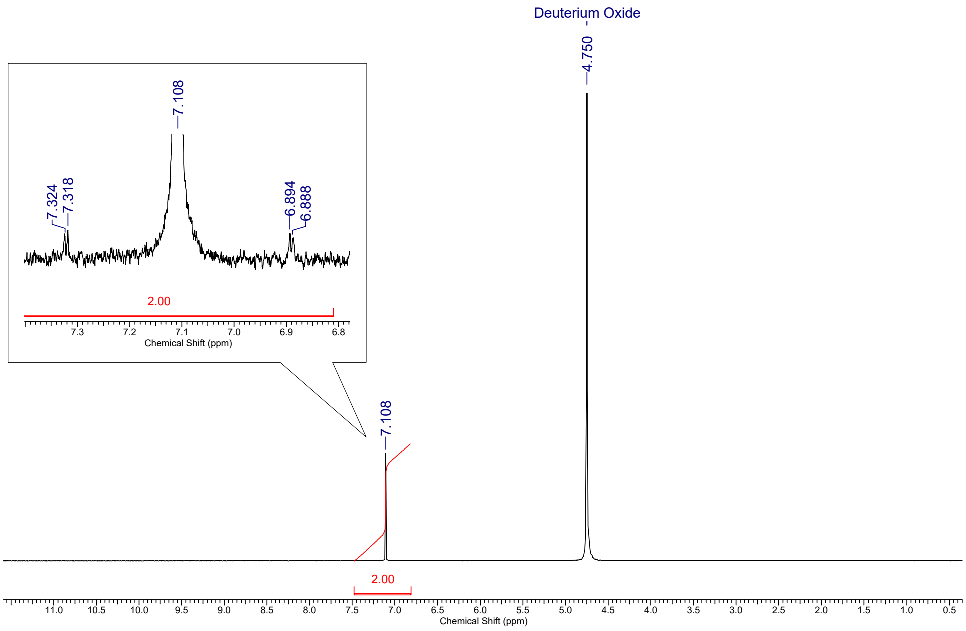

Figure S1. ^{1}H NMR spectrum (400 MHz, D_2O) of chelidonic acid.

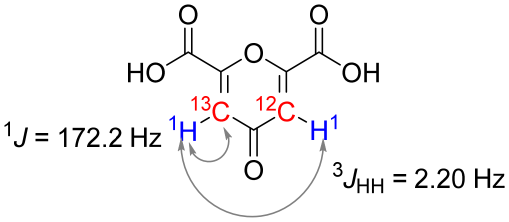

Figure S2. Spin-spin interaction constants in the minor isotopomer of chelidonic acid.

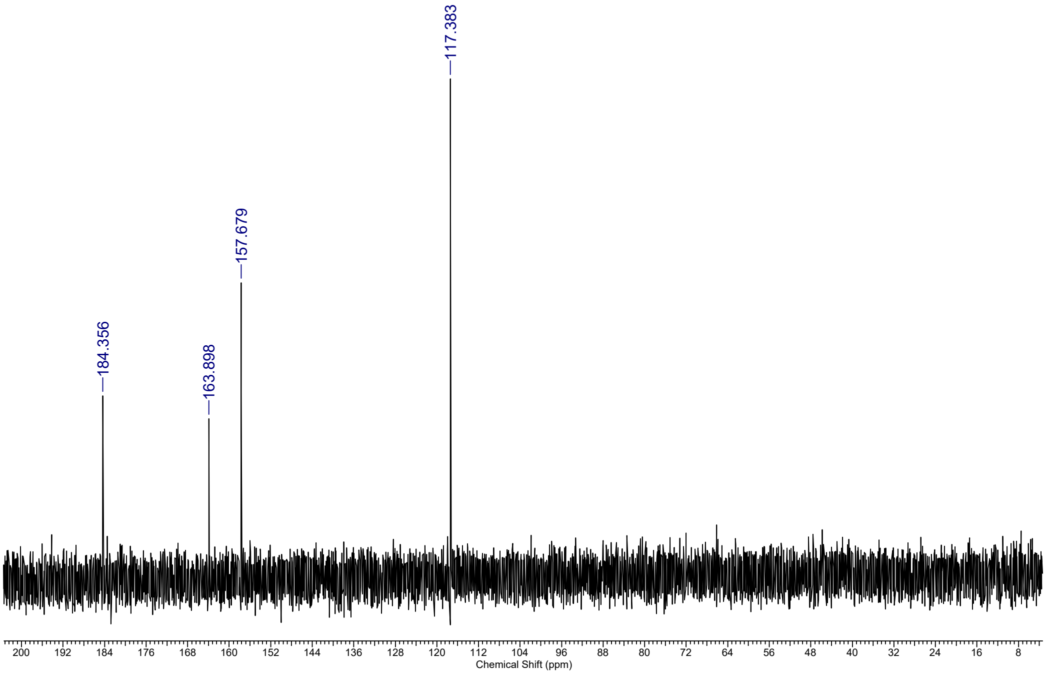

Figure S3. ^{13}C NMR spectrum (101 MHz, D_2O) of chelidonic acid.

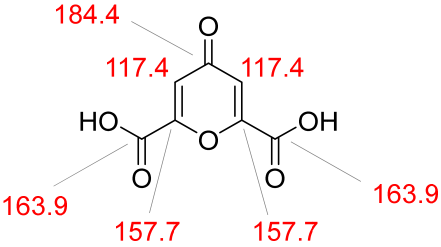

Figure S4. Assignment of signals in the ^{13}C NMR spectrum of chelidonic acid.

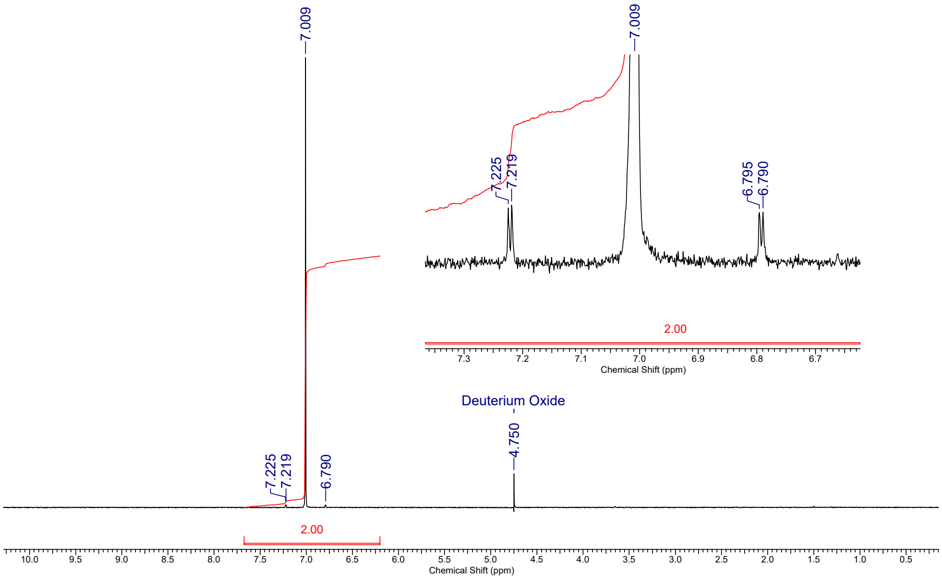

Figure S5. ^{1}H NMR spectrum (400 MHz, D_2O) of zinc chelidonate $[Zn(Chel)(H_2O)_4]_n$.

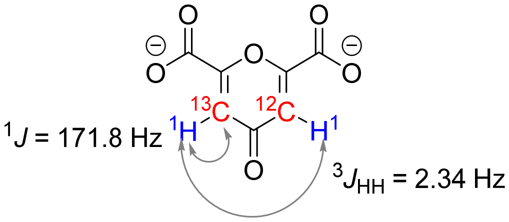

Figure S6. Spin-spin interaction constants in the minor isotopomer of the chelidonate ion.

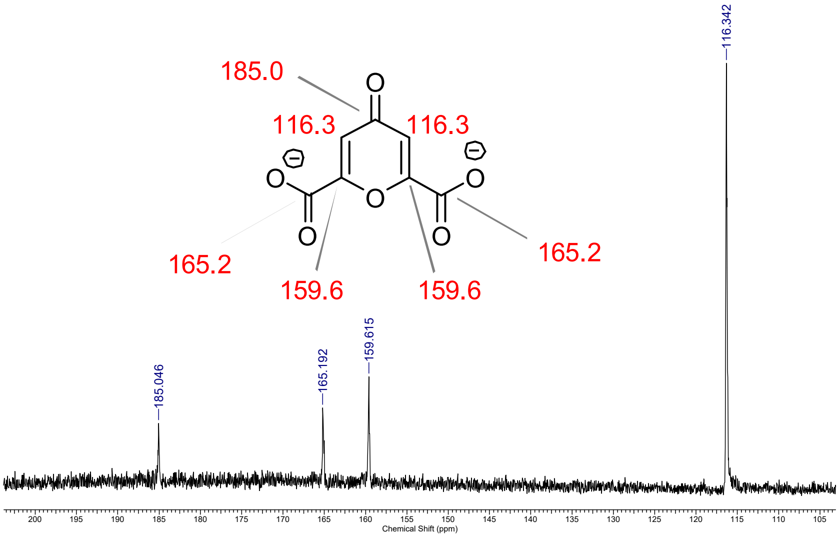

Figure S7. ^{13}C NMR spectrum (101 MHz, D_2O) of zinc chelidonate $[Zn(Chel)(H_2O)_4]_n$.

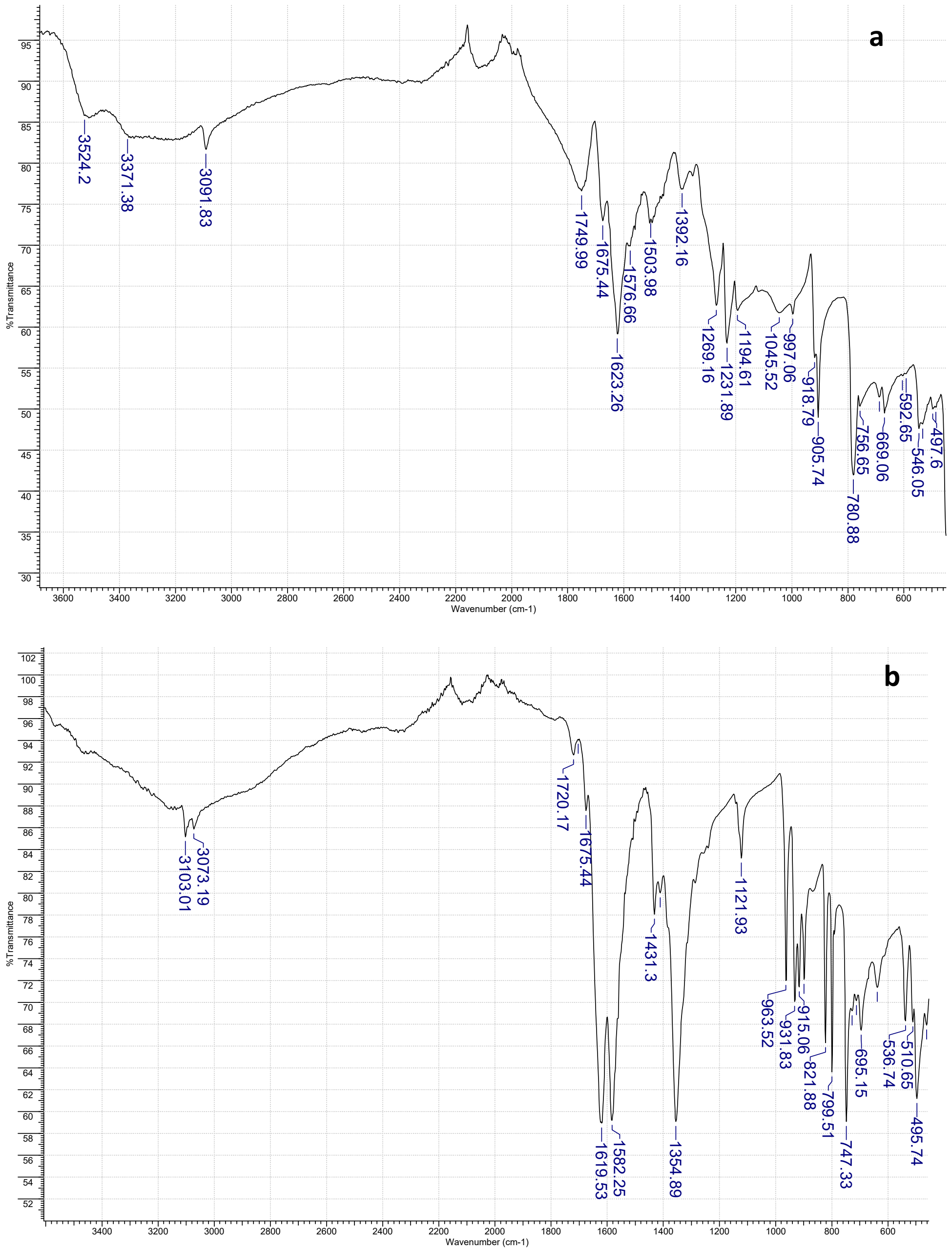

Figure S8. IR spectra of (**a**) chelidonic acid (**b**) zinc chelidonate $[Zn(Chel)(H_2O)_4]_n$

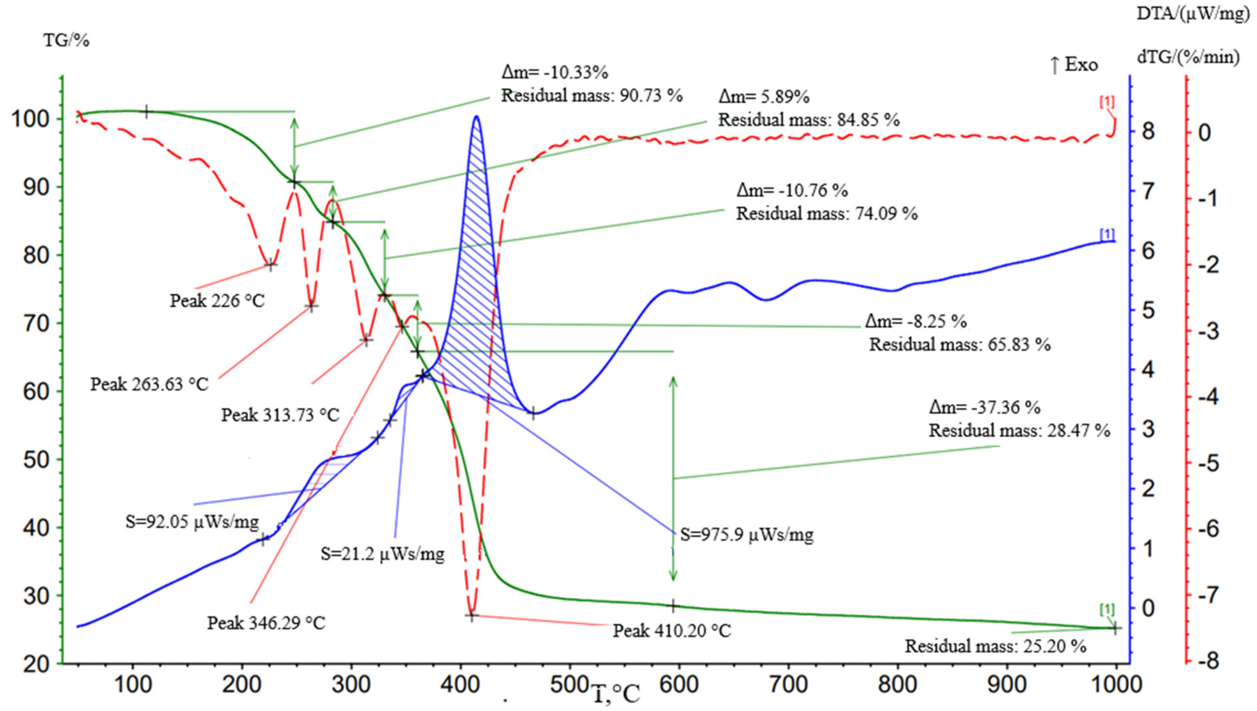

Figure S9. Thermal analysis data for zinc chelidonate.

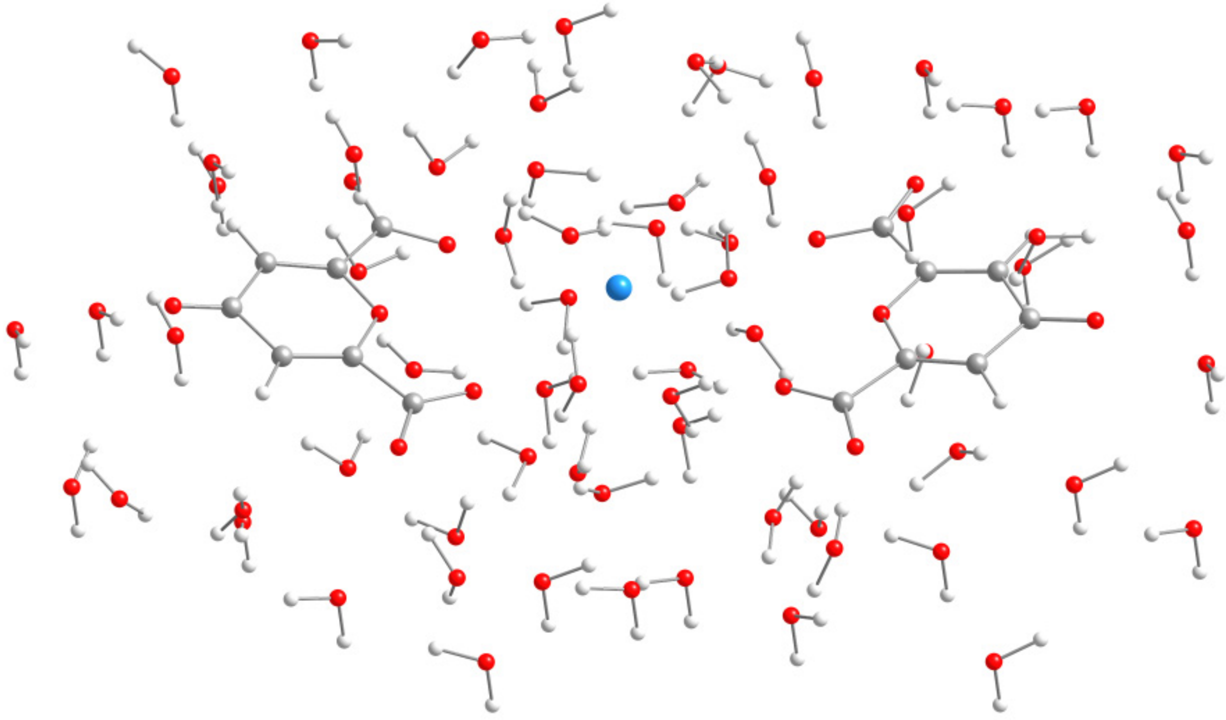

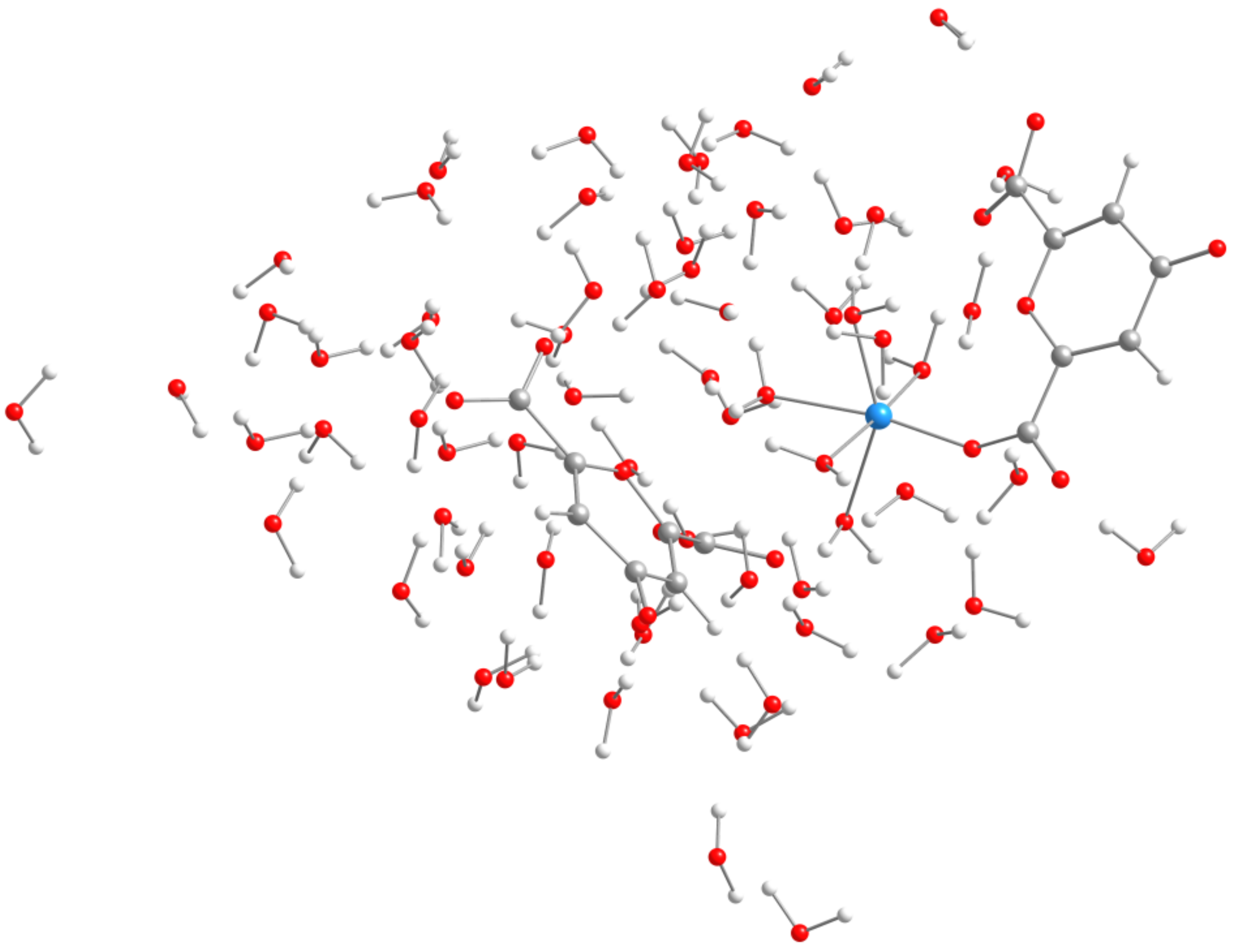

Figure S10. Initial and final results of molecular dynamics simulation (calculation at the GFN2-XTB level).

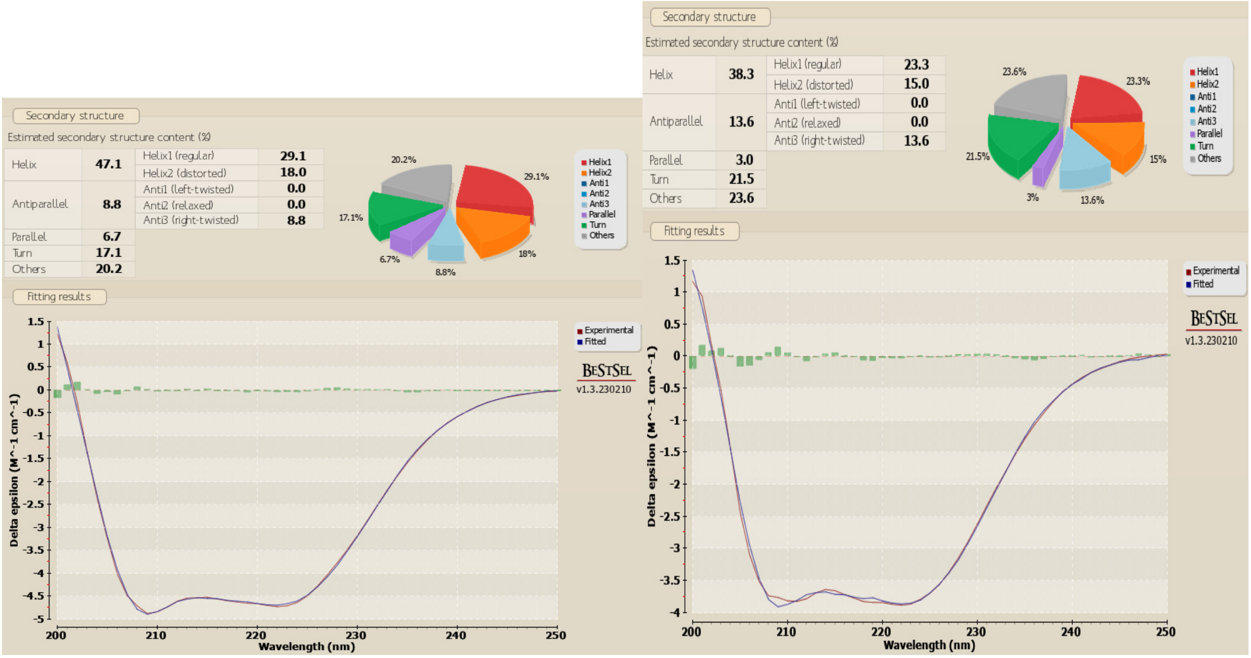

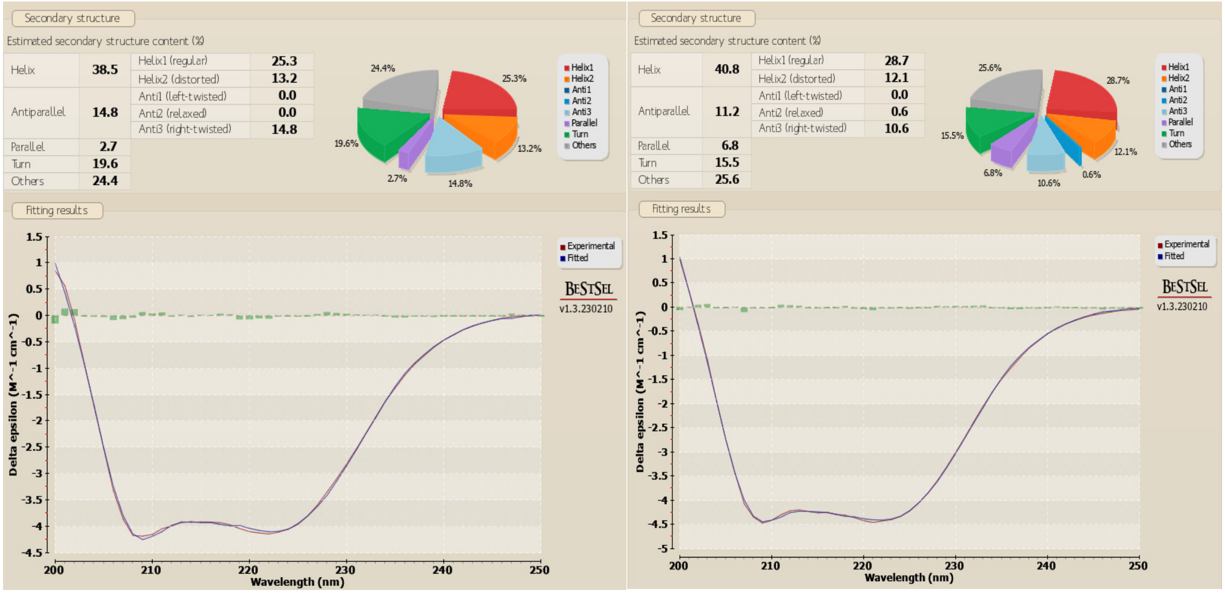

Figure S11. CD spectra in the presence and absence of zinc chelidonate.

Table S1. Composition of the zinc chelidonate complex according to the results of thermal analysis.

according to the thermogram			according to the X-ray diffraction		
ω (Zn), mass. %	ω (hel), mass. %	ω (H_2O), масс. %	ω (Zn), mass. %	ω (hel), mass. %	ω (H_2O), mass. %
20,25	56,37	18,43	20,34	56,99	22,55

Table S2. Crystallographic data, experimental parameters and refinements of the structure of zinc chelidonate.

Empirical formula	$C_7H_{10}O_{10}Zn$
Formula weight	319.52
Temperature/K	100(2)
Crystal system	triclinic
Space group	P-1
a/Å	5.0222(3)
b/Å	5.2447(4)
c/Å	9.9070(9)
α/°	94.470(7)
β/°	93.965(6)
γ/°	98.497(6)
Volume/Å^3	256.44(3)
Z	1
ρ_{calc}g/cm^3	2.069
μ/mm^{-1}	3.886
F(000)	162.0
Crystal size/mm^3	0.399 × 0.192 × 0.104
Radiation	Cu Kα (λ = 1.54184)
2Θ range for data collection/°	8.984 to 151.762
Index ranges	-6 ≤ h ≤ 6, -6 ≤ k ≤ 6, -12 ≤ l ≤ 12
Reflections collected	5279
Independent reflections	5279 [R_{int} = ?, R_{sigma} = 0.0078]

Data/restraints/parameters	5279/3/132
Goodness-of-fit on F^2	1.073
Final R indexes [$I>=2\sigma$ (I)]	R_1 = 0.0619, wR_2 = 0.1678
Final R indexes [all data]	R_1 = 0.0620, wR_2 = 0.1679
Largest diff. peak/hole / e $Å^{-3}$	1.72/-1.01

Table S3. Crystallographic distances between atoms.

Atom	Atom	Length/Å	Atom	Atom	Length/Å
Zn1	O5[1]	2.064(3)	C2	C3	1.520(14)
Zn1	O5	2.064(3)	O4	C3	1.311(11)
Zn1	O6[2]	2.078(4)	O4	C4	1.366(12)
Zn1	O6[3]	2.078(4)	O4	C5	1.291(12)
Zn1	O3	2.114(5)	C6	C3[4]	1.442(13)
Zn1	O3[1]	2.114(5)	C6	C4[4]	1.398(11)
O6	C1	1.196(13)	C6	C4	1.382(13)
O3	C2	1.206(12)	C6	C5	1.461(13)
O2	C2	1.212(16)	C3	C4	1.410(15)
C1	C5	1.511(13)	C4	C4[4]	1.34(2)
C1	O1	1.232(16)	C4	C5	1.398(14)

[1]-X,3-Y,1-Z; [2]-X,2-Y,1-Z; [3]+X,1+Y,+Z; [4]-X,2-Y,-Z

Table S4. Crystallographic angles between atoms.

Atom	Atom	Atom	Angle/°	Atom	Atom	Atom	Angle/°
O5[1]	Zn1	O5	180.0	C4	C6	C5	58.8(7)
O5[1]	Zn1	O6[2]	88.97(17)	C4[4]	C6	C5	116.1(8)
O5	Zn1	O6[2]	91.03(17)	O4	C3	C2	115.4(9)
O5[1]	Zn1	O6[3]	91.03(17)	O4	C3	C6[4]	118.8(8)
O5	Zn1	O6[3]	88.97(17)	O4	C3	C4	60.1(7)
O5	Zn1	O3	89.27(16)	C6[4]	C3	C2	125.7(9)
O5	Zn1	O3[1]	90.73(16)	C4	C3	C2	174.8(10)
O5[1]	Zn1	O3[1]	89.27(16)	C4	C3	C6[4]	58.7(6)
O5[1]	Zn1	O3	90.73(16)	O4	C4	C6	119.1(8)
O6[2]	Zn1	O6[3]	180.0	O4	C4	C6[4]	118.1(9)
O6[2]	Zn1	O3[1]	93.84(18)	O4	C4	C3	56.3(7)
O6[3]	Zn1	O3	93.84(18)	O4	C4	C5	55.7(7)
O6[2]	Zn1	O3	86.16(18)	C6	C4	C6[4]	122.5(9)
O6[3]	Zn1	O3[1]	86.16(18)	C6	C4	C3	174.0(11)
O3	Zn1	O3[1]	180.0	C6[4]	C4	C3	61.8(7)
C2	O3	Zn1	132.3(6)	C6	C4	C5	63.4(7)
O6	C1	C5	116.6(10)	C4[4]	C4	O4	174.9(14)
O6	C1	O1	125.6(10)	C4[4]	C4	C6[4]	60.7(8)
O1	C1	C5	117.4(10)	C4[4]	C4	C6	61.9(9)
O3	C2	O2	126.3(10)	C4[4]	C4	C3	122.2(13)
O3	C2	C3	116.5(10)	C4[4]	C4	C5	125.0(14)
O2	C2	C3	116.9(10)	O4	C5	C1	116.3(9)
C3	O4	C4	63.5(7)	O4	C5	C6	118.7(8)

Atom	Atom	Atom	Angle/°	Atom	Atom	Atom	Angle/°
C5	O4	C4	63.4(7)	O4	C5	C4	60.9(7)
C4[4]	C6	C3[4]	59.5(6)	C6	C5	C1	125.0(9)
C4	C6	C3[4]	116.8(8)	C4	C5	C1	176.3(11)
C4	C6	C4[4]	57.5(9)	C4	C5	C6	57.8(7)

[1]-X,3-Y,1-Z; [2]+X,1+Y,+Z; [3]-X,2-Y,1-Z; [4]-X,2-Y,-Z

Table S5. Hydrogen bond parameters.

D	H	A	d(D-H)/Å	d(H-A)/Å	d(D-A)/Å	D-H-A/°
O6	H6A	O2[1]	0.87	2.05	2.757(11)	137.7
O3	H3A	O1[2]	0.87	1.89	2.747(10)	167.2
O3	H3B	O4	0.87	1.77	2.636(6)	170.4
O5	H5A	O3[3]	0.90(4)	1.95(4)	2.844(6)	172(6)

[1]1+X,-1+Y,+Z; [2]-1+X,1+Y,+Z; [3]-1-X,3-Y,1-Z

Table S6. Geometric parameters of zinc coordination nodes in molecular associates of $ZnChel(H_2O)_5{\cdot}H_2O$ и $[Zn(H_2O)_6]^{2+}{\cdot}Chel^{2-}$ compositions (calculation at the ωB97X-3c level).

Associate	Connection	Length, Å	Corner	Size, °
$ZnChel(H_2O)_5{\cdot}H_2O$	Zn1 – O12	2.004	O12 – Zn1 – O17	95.6
	Zn1 – O17	2.095	O12 – Zn1 – O20	101.3
	Zn1 – O20	2.070	O12 – Zn1 – O23	94.3
	Zn1 – O23	2.176	O12 – Zn1 – O29	84.1
	Zn1 – O29	2.179	O12 – Zn1 – O32	169.8
	Zn1 – O32	2.185	O17 – Zn1 – O20	87.7
			O17 – Zn1 – O23	167.7
			O17 – Zn1 – O29	90.6
			O17 – Zn1 – O32	86.0
			O20 – Zn1 – O23	85.9
			O20 – Zn1 – O29	168.1
			O20 – Zn1 – O32	88.3
			O23 – Zn1 – O29	83.2
			O23 – Zn1 – O32	83.0
			O29 – Zn1 – O32	85.9
$[Zn(H_2O)_6]^{2+}{\cdot}Chel^{2-}$	Zn1 – O17	2.145	O17 – Zn1 – O20	172.3
	Zn1 – O20	2.122	O17 – Zn1 – O23	88.5
	Zn1 – O23	2.152	O17 – Zn1 – O26	86.3
	Zn1 – O26	2.128	O17 – Zn1 – O29	96.7
	Zn1 – O29	2.070	O17 – Zn1 – O32	84.9
	Zn1 – O32	2.110	O20 – Zn1 – O23	89.2
			O20 – Zn1 – O26	86.4
			O20 – Zn1 – O29	90.6
			O20 – Zn1 – O32	97.3
			O23 – Zn1 – O26	90.3
			O23 – Zn1 – O29	87.9
			O23 – Zn1 – O32	173.4
			O26 – Zn1 – O29	176.5

			O26 – Zn1 – O32<br>O29 – Zn1 – O32	89.3<br>92.9
